# Thymol carbamates bearing cyclic amines as potent and selective BuChE inhibitors alleviate memory impairments for Alzheimer’s disease therapy

**DOI:** 10.1080/14756366.2026.2623314

**Published:** 2026-02-06

**Authors:** Chengyao Wu, Yulu Ding, Xiaodan Liu, Shan Gao, Xiaoqing Wang, Wenjian Tang

**Affiliations:** ^a^ The First People’s Hospital of Chuzhou, The Affiliated Chuzhou Hospital of Anhui Medical University, Chuzhou, China; ^b^ School of Pharmacy, Anhui Medical University, Hefei, China

**Keywords:** Thymol, carbamate, butyrylcholinesterase, pseudo-irreversible, Alzheimer’s disease

## Abstract

Thymol, an isomer of carvacrol, exhibits anti-A*β* activity. Thymol carbamates were designed, and their inhibition on cholinesterase (ChE) activity was assessed and analysed, among them, **TC-4**, **TC-6**, **H4** and **H5** bearing cyclic amines exhibited nanomolar inhibitory activity with IC_50_ values of 13, 3.6, 47, and 12 nM. **TC-6** bearing a piperidinyl moiety demonstrated nanomolar *h*BuChE inhibition (IC_50_ = 3.6 nM), >2,500-fold selectivity over *h*AChE, and pseudo-irreversible kinetics (*K*_d_ = 0.25 μM, *k*_2_ = 0.98 min^−1^). **TC-6** exhibited low cytotoxicity, crossed the blood-brain barrier, and protected neurons against H_2_O_2_-induced damage. In A*β*_1-42_-induced AD mice, **TC-6** (10 mg/kg) greatly enhanced cognitive abilities in MWM tests, reduced brain A*β* levels, and restored hippocampal neuron density. These results highlight **TC-6** as a potent, brain-penetrant BuChE inhibitor with therapeutic potential for AD.

## Introduction

Dementia is an age-related neurodegenerative disorder affecting more than 55 million people worldwide, with Alzheimer’s disease (AD) accounting for approximately 60–80% of all cases. AD is characterised by progressive neurodegeneration and cognitive decline.^1–3^ Several hypotheses have been proposed to explain the pathogenesis of AD, including the amyloid-*β* cascade hypothesis, tau hyperphosphorylation hypothesis, cholinergic hypothesis, and neuroinflammation hypothesis.^4–6^ Currently approved therapeutic agents for AD include three cholinesterase inhibitors – donepezil, rivastigmine and galantamine – and one N-methyl-D-aspartate (NMDA) receptor antagonist, memantine,^7^^,^^8^ as well as two monoclonal antibodies.^9^^,^^10^ However, due to the complex and multifactorial pathophysiology of AD, these treatments are unable to halt or reverse disease progression.In recent years, accumulating evidence has identified misfolded amyloid-β (A*β*) and hyperphosphorylated tau as central pathological hallmarks of AD.^11^ Among the approved therapeutic strategies, modulation of the cholinergic system through cholinesterase inhibition remains one of the most effective approaches for improving cognitive symptoms.^12–19^

Impairment of acetylcholine (ACh) signalling is strongly correlated with the hallmark features of AD, including cognitive deficits and memory deterioration.^20^^,^^21^ ACh is hydrolysed by cholinesterases (ChEs), which are mainly classified into acetylcholinesterase (AChE) and butyrylcholinesterase (BuChE). AChE primarily catalyses the hydrolysis of acetylcholine, whereas BuChE has traditionally been regarded as a less prominent pseudocholinesterase. However, in patients with late-stage AD, AChE levels are markedly reduced, leading to a substantial loss of its enzymatic activity. In contrast, BuChE levels do not decline but instead increase, thereby assuming a predominant role in ACh hydrolysis.^22–26^ AChE and BuChE share approximately 65% sequence homology,^27^ and differences in residues within the acyl-binding pocket are critical for the design of highly selective BuChE inhibitors.^28–30^

Rivastigmine, which contains a carbamate moiety as its core structural feature, represents a clinically approved carbamate-based AChE inhibitor used in the treatment of AD.^31^ The carbamate moiety interacts with key residues within the acyl-binding pocket, including Tyr120, His438, and Ser198.^32^ Incorporation of this fragment into novel compounds facilitates targeted binding to the acyl-binding pocket, resulting in covalent inhibition of BuChE. Therefore, the carbamate fragment plays a crucial role in BuChE inhibition.^33^^,^^34^ Accordingly, the synthesis of novel carbamates represents a promising strategy for the development of therapeutic agents for AD.

Thymol is a naturally occurring phenolic compound primarily isolated from thyme.^35^ It has been widely reported to exhibit anti-inflammatory,^36–39^ antioxidant.^40–44^ and antibacterial activities.^45–49^ In addition, accumulating evidence suggests that thymol can reduce oxidative stress in the brain by decreasing amyloid-β (A*β*) accumulation. Moreover, thymol has been shown to markedly downregulate tumour necrosis factor-α (TNF-α), a key pro-inflammatory mediator, thereby improving learning and memory performance as well as other physiological characteristics in AD mouse models; however, the underlying mechanisms remain to be fully elucidated.^50^ Furthermore, thymol-derived 2-aminothiol and sulphonic acid derivatives have demonstrated notable inhibitory activity against both AChE and BuChE, although they lack significant selectivity towards either enzyme.^51^

**Figure 1.**
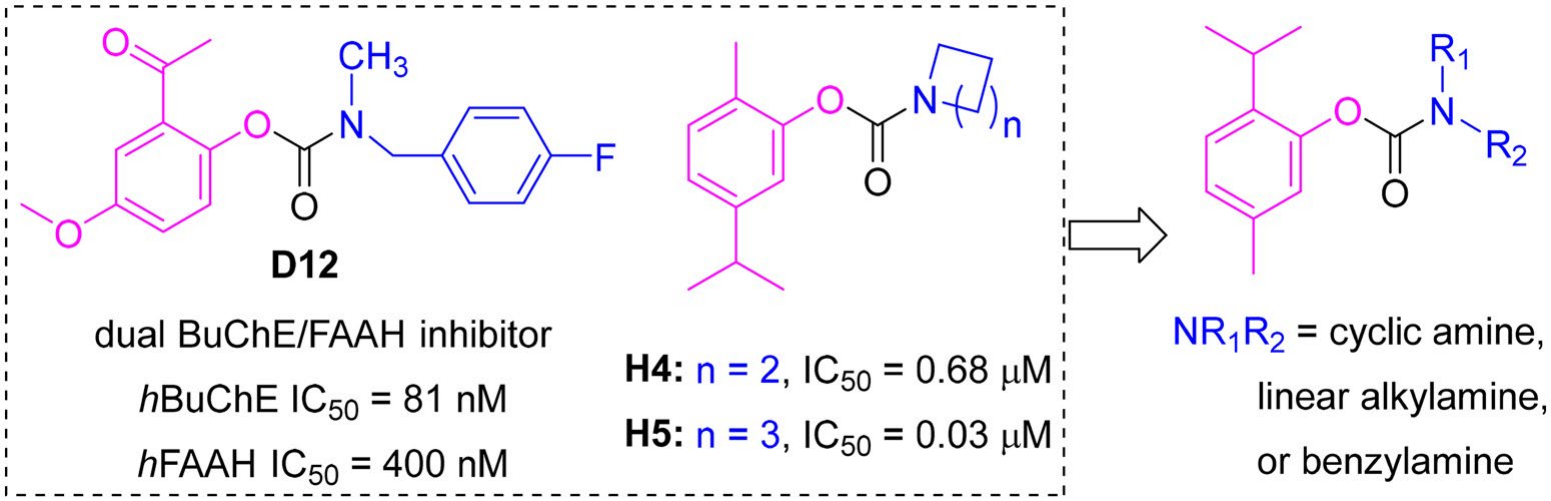
The overall design strategy for this research.

Recently, paeonol-derived carbamate **D12**, originating from natural phenols, was identified as a dual BuChE and fatty acid amide hydrolase (FAAH) inhibitor and exhibited significant neuroprotective effects in an Alzheimer’s disease mouse model.^51^ Further studies revealed that carvacrol carbamates **H4** and **H5** function as selective BuChE inhibitors, with IC_50_ values of 0.68 and 0.03 μM, respectively. Notably, the cyclic amine cores of **H4** and **H5** were found to confer favourable brain penetration, as indicated by structure–tissue exposure/selectivity relationship analyses of cannabidiol carbamates.^52^^,^^53^ While carbamates of natural phenols such as carvacrol and paeonol have been explored as BuChE inhibitors, thymol-based carbamates bearing cyclic amine moieties remain largely unexplored as selective, pseudo-irreversible BuChE inhibitors. This study designed, synthesised, and evaluated a novel series of thymol carbamates, leading to the identification of **TC-6** exhibiting nanomolar BuChE inhibition, exceptional selectivity (>2,500-fold over AChE), and favourable brain penetration. These findings introduce a new structural class with strong potential for AD therapy (Figure 1).

## Results and discussion

### Chemistry

**Scheme 1.**
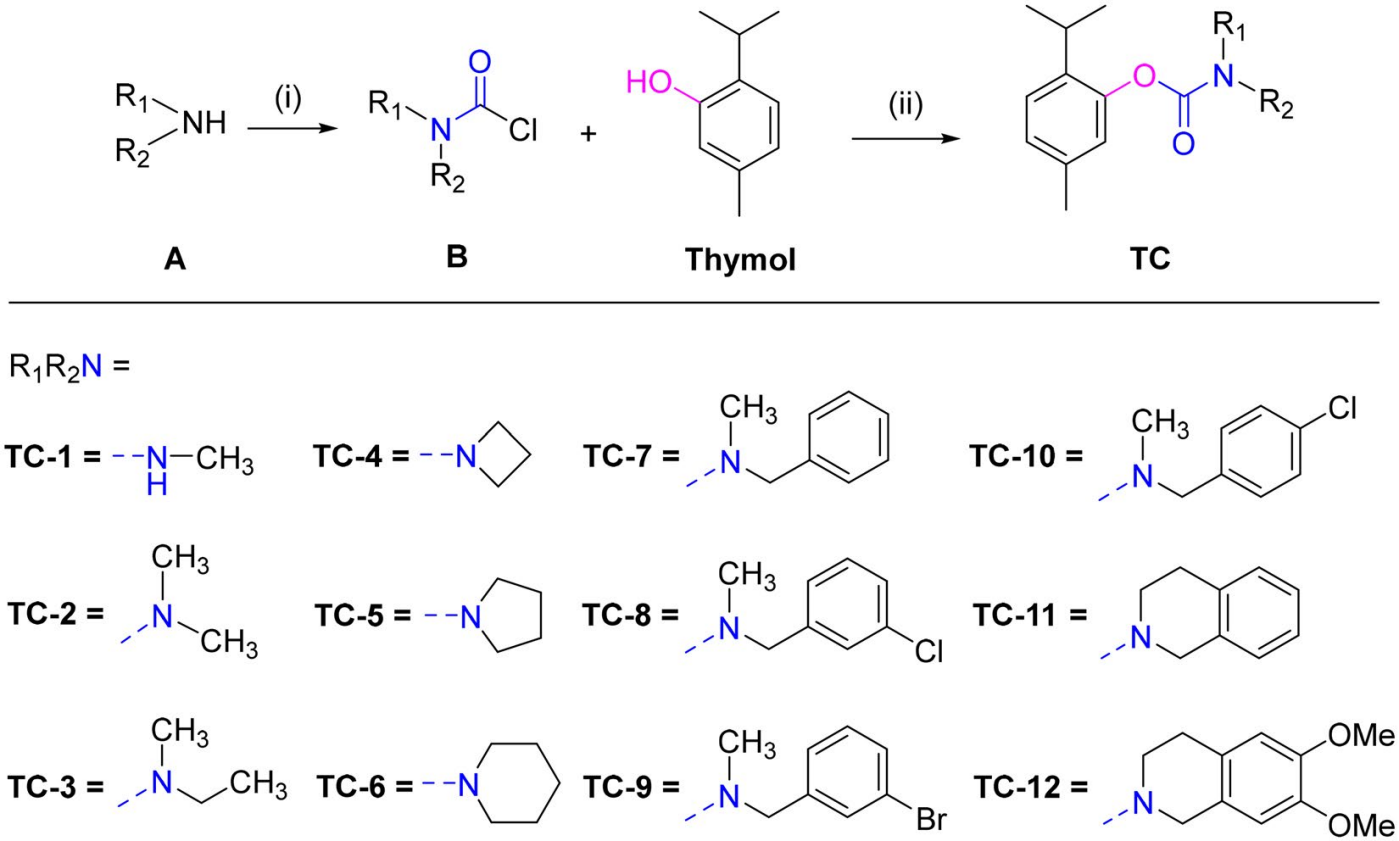
Chemical structures of thymol carbamates **TC-1–TC-12**. ^a^Reagents and conditions: (i) Tri-phosgene, NaHCO_3_, CH_2_Cl_2_, –10**–**0 °C, 6 h; (ii) DMAP, K_2_CO_3_, CH_3_CN, 60**–**65 °C.

Thymol carbamates were prepared using a published method for natural phenol carbamate synthesis.^52^ Under alkaline conditions, triphosgene reacts with either primary or secondary amines (**A**) to yield aminocarbonyl chloride (**B**). Thymol, acting as an effective nucleophile, is then introduced under basic conditions to form thymol carbamates. Typically, the aminocarbonyl chloride can be directly utilised without separation, ensuring a combined yield of both steps that is at least 50%. The final products **TC-1**–**TC-12** were purified using rapid column chromatography on silica gel in Scheme 1. The structures of synthesised compounds were confirmed by high-resolution mass spectrometry (HRMS), as well as ^1^H and ^13^C nuclear magnetic resonance (NMR) spectroscopy (Supporting Information). **TC**-series represents a structurally innovative class of BuChE inhibitors, wherein thymol is linked via a carbamate bridge to various cyclic amines. This design not only retains the inherent anti-Aβ and antioxidant properties of thymol but also enhances steric and electronic complementarity with the BuChE active site, resulting in unprecedented inhibitory potency and selectivity. Notably, **TC-6**, featuring a piperidine ring, emerges as the most potent analogue reported to date within this scaffold. The purity of **TC-6** determined by HPLC was 98.9% (see Supplementary Table S1).

### Inhibition of AChE and BuChE activities

Using Ellman’s assay, the TC series of compounds was screened for inhibitory activity against electric eel acetylcholinesterase (EeAChE) and equine butyrylcholinesterase (eqBuChE). The half-maximal inhibitory concentrations (IC_50_) were determined and benchmarked against donepezil as the reference standard. Table 1 presents the complete IC_50_ profile for all tested compounds against both enzymatic targets.

**Table 1.** Inhibitory effects of compounds **TC-1–TC-12**.^a^

Compound	AChE^b^	BuChE^c^
	(IC_50_, µM or % inhibition at 20 µM)	
**TC-1**	11.7 ± 1.7%	2.75 ± 0.72
**TC-2**	14.7 ± 1.9%	0.29 ± 0.031
**TC-3**	17.9 ± 2.3%	0.51 ± 0.11
**TC-4**	*na*	0.0028 ± 0.0003
**TC-5**	30.1 ± 3.0%	0.026 ± 0.002
**TC-6**	*na*	0.0063 ± 0.0003
**TC-7**	13.7 ± 1.1%	5.10 ± 0.63
**TC-8**	14.2 ± 4.1%	3.84 ± 0.60
**TC-9**	17.3 ± 3.6%	2.13 ± 0.68
**TC-10**	23.5 ± 1.2%	3.91 ± 0.49
**TC-11**	*na*	2.79 ± 0.27
**TC-12**	11.4 ± 1.0%	4.04 ± 0.53
**H4**	*na*	1.61 ± 0.44
**H5**	8.3%	0.031 ± 0.014
Donepezil	0.032 ± 0.0072	9.21 ± 1.09
Rivastigmine	13.7 ± 1.1	0.067 ± 0.023

^a^Each IC_50_ value was calculated based on the average ± SEM of three separate trials; On EeAChE and eqBuChE. ^b^Acetylcholinesterase derived from electric eel; ^c^Butyrylcholinesterase derived from horse serum; ^d^*na*, Inhibition of EeAChE and eqBuChE was not observed at 20 µM.

**Figure 2.**
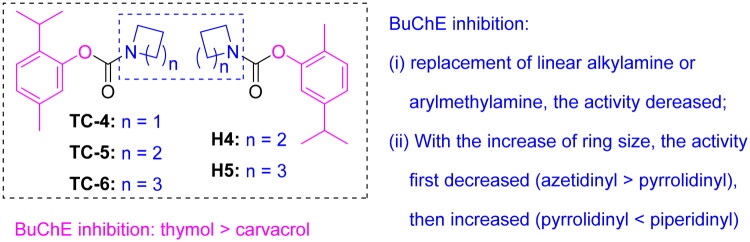
SARs of thymol carbamates as BuChE inhibitors.

According to the data presented in Table 1 and Figure 2, incorporating an amine unit appears to influence the selectivity and inhibitory activity of thymol carbamates towards AChE and BuChE. All thymol carbamates exhibited high selectivity for *h*BuChE over *h*AChE, with most compounds showing micromolar-level inhibition of BuChE. Notably, compounds **TC-4**, **TC-6**, **H4** and **H5** demonstrated nanomolar inhibition, with IC_50_ values of 13, 3.6, 47, and 12 nM, respectively.

Recent study revealed that the phenolic unit of carbamate plays an important role in ChE inhibitory activity.^18^^,^^52^ Further structure-activity relationship (SAR) analysis showed that: (i) BuChE inhibitory effect of phenolic core (thymol vs. carvacrol), the thymol were close or superior to carvacrol (**TC-1**, 2.75 μM ≈ **H1**, 2.96 μM for –NHMe, **TC-2**, 0.29 μM ≈ **H2**, 0.59 μM for –NMe_2_, **TC-3**, 0.51 μM ≈ **H3**, 0.78 μM for –N(Me)Et, **TC-8**, 3.84 μM ≈ **H8**, 1.13 μM for –NMeCH_2_phenyl-3-Cl, **TC-9**, 2.13 μM ≈ **H7**, 3.84 μM for –NMeCH_2_phenyl-3-Br; **TC-5**, 0.026 μM > **H4**, 1.61 μM for –N(CH_2_)_4_, **TC-6**, 0.0063 μM > **H5**, 0.031 μM for –N(CH_2_)_5_); (ii) BuChE inhibitory effect of amine substituent, although phenol carbamates containing benzylamine were reported to be potent BuChE inhibitory activity,^52^ thymol carbamates containing benzylamine only had micromolar BuChE inhibitory activity (IC_50_ values of 2–6 μM for **TC-7–TC-10**), and those containing a tetrahydroisoquinoline showed similar activity (**TC-11**, 2.79 μM, **TC-12**, 4.04 μM); (iii) influence of ring size within cyclic amines, with the increase of ring size, the activity first decreased (**TC-4** of azetidinyl > **TC-5** of pyrrolidinyl) and then increased (**TC-5** of pyrrolidinyl < **TC-6** of piperidinyl), similarly, for carvacrol carbamates, piperidinyl (**H5**) was greater than pyrrolidinyl (**H4**) (Figure 2).

### Inhibition of hBuChE and hAChE

To assess compound efficacy and selectivity, the inhibitory activities of the synthesised compounds against *h*BuChE and *h*AChE were evaluated. As shown in Table 2, compounds **TC-2**, **TC-4**, **TC-5**, **TC-6**, **H4** and **H5** exhibited IC_50_ values of 2.67, 0.013, 0.14, 0.0036, 0.047, and 0.013 μM against *h*BuChE, respectively, which were lower than those of the positive control donepezil, indicating superior inhibitory potency. Furthermore, cyclic amine–bearing thymol/carvacrol carbamates **TC-4**, **TC-5**, **TC-6**, **H4** and **H5** were identified as highly selective for *h*BuChE inhibitors, among which thymol carbamate **TC-6** exhibited the most favourable IC_50_ value.

**Table 2.** Inhibitory potency towards *h*BuChE and *h*AChE.^a^

Compound	IC_50_ (μM) or inhibition rate (%)	
	*h*AChE	*h*BuChE
**Donepezil**	0.015 ± 0.033	9.32 ± 0.17
**Rivastigmine**	6.9%	5.13 ± 0.08
**TC-2**	*na* ^b^	2.67 ± 0.15
**TC-4**	*na*	0.013 ± 0.003
**TC-5**	17%	0.14 ± 0.06
**TC-6**	*na*	0.0036 ± 0.0012
**H4**	15%	0.047 ± 0.050
**H5**	*na*	0.012 ± 0.004

^a^Each IC_50_ represents the mean ± SEM of three or more independent trials. ^b^*na*, no activity.

### Molecular docking study

**Figure 3.**
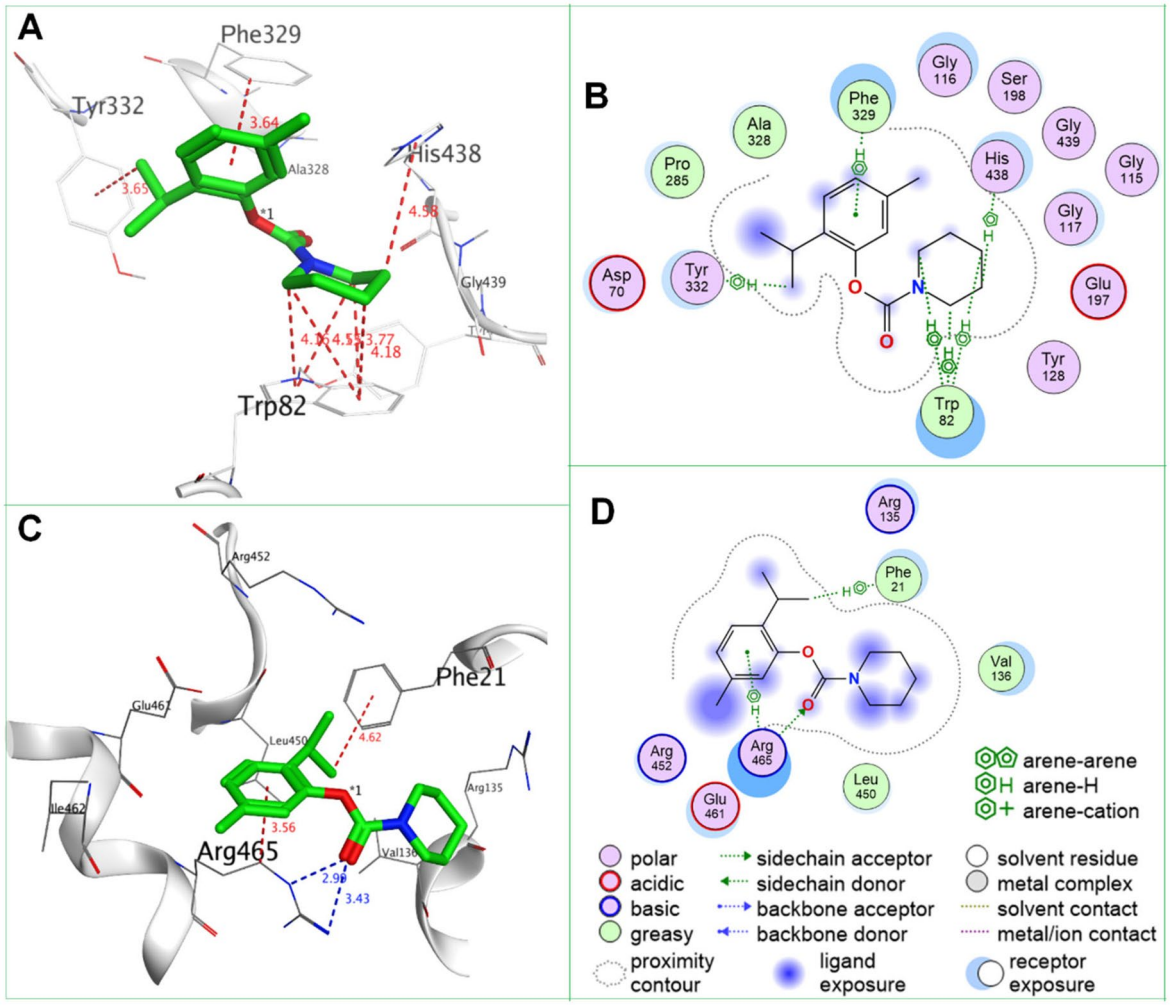
3D mode (A) and 2D mode (B) of interactions of **TC-6** with receptor *h*BuChE (PDB: 1XLU); 3D mode (C) and 2D mode (D) of interactions of **TC-6** with receptor *h*BuChE (PDB: 4BDS). Shades of green and vivid yellow are used to represent **TC-6**, while π–π interactions and hydrogen bonds are denoted by purple and green dashed lines, respectively. key residues are marked in blue.

To elucidate the binding modes and interactions of thymol carbamates with human butyrylcholinesterase (*h*BuChE), the active compounds were docked into the crystal structures of diisopropyl fluorophosphate (DFP)-aged *h*BuChE and tacrine-bound *h*BuChE (PDB IDs: 1XLU and 4BDS). The docking results indicated that compounds bearing five-membered aliphatic cyclic amines exhibited less favourable predicted docking scores and weaker interactions, which was consistent with their lower bioactivity. In contrast, compounds containing six-membered rings, such as **TC-6**, displayed more favourable interactions with both 1XLU and 4BDS (Table 3). When the amine substituent was kept constant, the phenolic scaffold also influenced binding behaviour. In the 1XLU system, **TC-6** formed hydrogen-bond interactions involving the phenyl ring and Phe329, its isopropyl group and Tyr332, and its piperidine ring and both Tyr82 and His438. In the 4BDS system, hydrogen bonds were observed between the carbonyl oxygen and Arg465, as well as between the phenyl ring and Arg465, and between the isopropyl group and Phe21 (Figure 3). These findings suggest that BuChE inhibition is strongly modulated by both the nature and position of the substituents.

**Table 3.** Docking score of thymol carbamates and *h*BuChE (PDB: 1XLU and 4BDS).

Compd.	1XLU	4BDS
**TC-4**	‒5.8037	‒4.8280
**TC-5**	‒5.7894	‒5.0844
**TC-6**	‒6.2359	‒5.4342
**H-4**	‒6.4762	‒5.2620
**H-5**	‒6.6795	‒5.2034

### Kinetic study of eqBuChE inhibition

**Figure 4.**
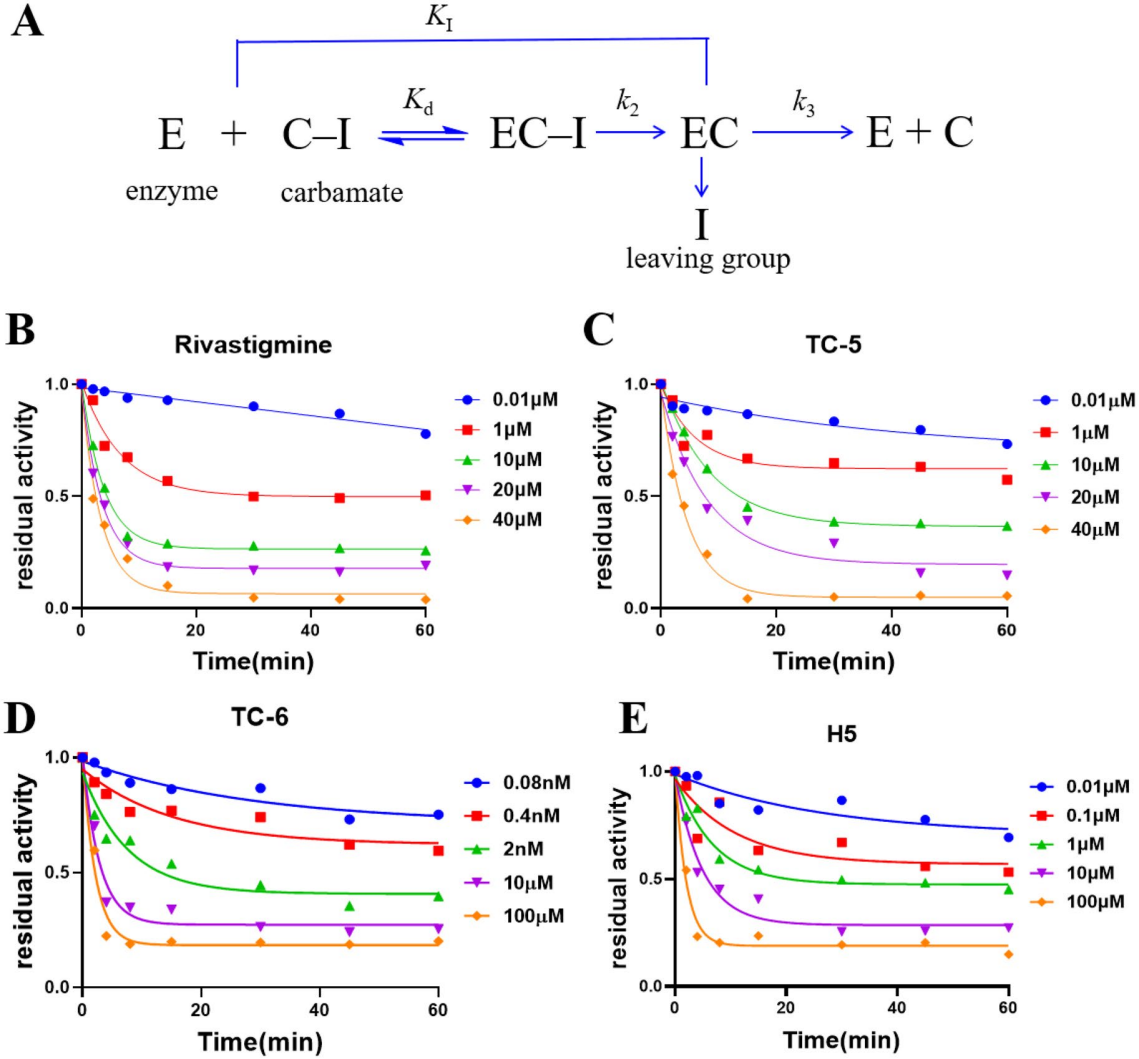
Pseudo-irreversible inhibition of eqBuChE by carbamate inhibitors. **(A)** Schematic illustration of pseudo-irreversible inhibition of enzyme E by carbamate inhibitors (C–I). **(B–E)** Time- and concentration-dependent carbamylation of eqBuChE by rivastigmine (B), **TC-5** (**C**), **TC-6** (**D**), and **H5** (**E**).

As shown in Figure 4(A), pseudo-irreversible enzyme inhibition can be described by three sequential stages. The pseudo-irreversible inhibition of equine butyrylcholinesterase (eqBuChE) by natural phenol carbamates **TC-5**, **TC-6** and **H5** was investigated.

In the first stage, a reversible enzyme–inhibitor complex (EC–I) is formed between cholinesterase (E) and a carbamyl-containing compound (C–I). This step is characterised by the dissociation constant *k*_d_ (μM), which reflects the equilibrium binding affinity between the enzyme and inhibitor. In the second stage, the enzyme cleaves the carbamate ester bond within the EC–I complex, leading to transfer of the carbamyl group to the enzyme and release of the inhibitor, thereby forming the carbamylated enzyme (EC). The rate of this process is governed by the carbamylation rate constant *k*_2_ (min^−1^), and the overall carbamylation efficiency is described by *k*_I_ (μM^−1 ^min^−1^), defined as *k*_2_/*k*_d_.

In the third stage, water molecules gradually hydrolyse the covalent bond between the enzyme and the carbamyl group, allowing slow recovery of enzymatic activity. This decarbamoylation process is characterised by the rate constant *k*_3_ (min^−1^) and occurs under conditions where the inhibitor concentration is well below *k*_d_, rendering the EC–I complex negligible. Typically, *k*_3_ is several orders of magnitude smaller than *k*_2_, reflecting the high hydrolytic stability of the carbamylated enzyme. Detailed kinetic derivations and fitting procedures are provided in the Supporting Information.

As shown in Figure 4(B–E) and summarised in Table 4, **TC-5**, **TC-6** and **H5** exhibited pseudo-irreversible inhibition profiles similar to that of rivastigmine. Although **TC-6** displayed a *k*_d_ value comparable to those of **H5** and **TC-5**, it exhibited a substantially higher carbamylation rate constant (*k*_2_), enabling rapid conversion of the reversible enzyme–inhibitor complex into a covalently modified inactive form. Moreover, the relatively slow decarbamoylation rate (*k*_3_) suggests prolonged persistence of the carbamylated enzyme prior to activity recovery. Overall, the sustained inhibitory effect of **TC-6** does not arise from enhanced initial binding affinity, but rather from coordinated modulation of carbamylation and enzyme reactivation kinetics. This kinetic profile preserves reversibility of inhibition while potentially enabling more durable pharmacological efficacy at lower exposure levels.

**Table 4.** BuChE carbamoylation and decarbamoylation kinetics.^e^

Compound	*k*_d_ (μM)^a^	*k*_2_ (min^‒1^)^b^	*k*_I_ (μM^‒1^ min^‒1^)*10^‒3c^	*k*_3_ (min^‒1^)^d^
**TC-5**	0.15 ± 0.01	0.11 ± 0.01	0.27 ± 0.14	3.93 ± 0.47
**TC-6**	0.25 ± 0.07	0.98 ± 0.60	0.07 ± 0.03	0.48 ± 0.15
**H5**	0.18 ± 0.19	0.040 ± 0.080	0.33 ± 0.06	2.30 ± 0.78
Rivastigmine	1.74 ± 0.14	0.38 ± 0.04	1.35 ± 0.19	5.93 ± 0.38

^a^Observed enzyme–inhibitor binding affinity at equilibrium; ^b^Carbamylation rate constant; ^c^Overall carbamylation rate constant; ^d^Decarbamoylation rate; ^e^Values are expressed as mean ± SEM from ≥ 3 independent experiments.

### Evaluation of cytotoxicity

**Figure 5.**
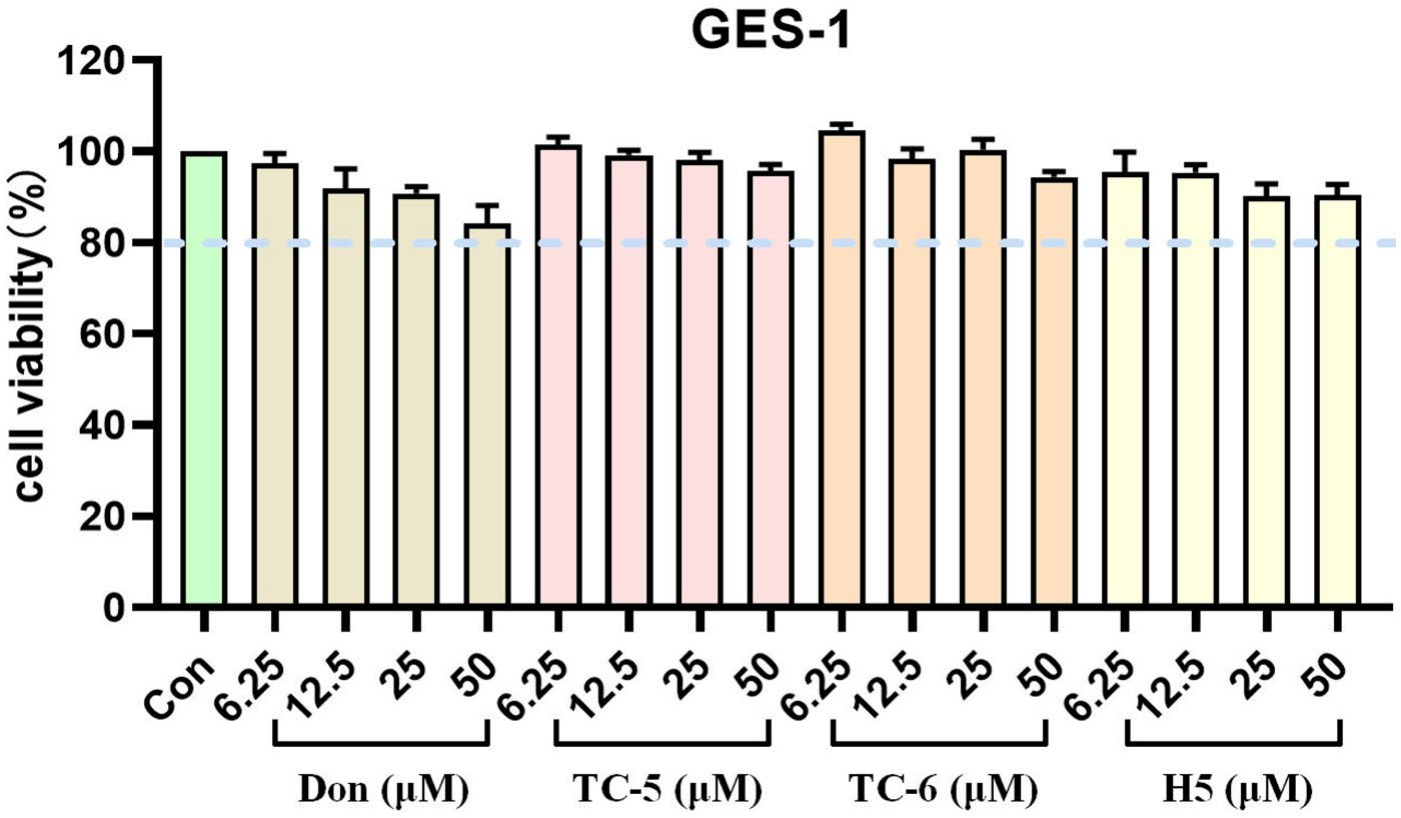
The cytotoxicity of donepezil, **TC-5**, **TC-6** and **H5** towards GES-1 cells was tested at 5–50 μM for 24 h. The control group remained untreated. Data were presented as the percentage of cell viability relative to the untreated cells, depicted as mean ± standard deviation.

To evaluate the *in vitro* cytotoxicity of the synthesised compounds, GES-1 gastric mucosal epithelial cells were selected. The compounds were tested at concentrations of 6.25, 12.5, 25, and 50 μM, and the effects of donepezil, **TC-5**, **TC-6**, and **H5** on cell viability were simultaneously assessed, as shown in Figure 5. After 24 h of exposure to **TC-5**, **TC-6**, and **H5** at 50 μM, GES-1 cell viability remained largely unchanged, with viability values of 97.0%, 94.4%, and 90.3%, respectively. In contrast, donepezil at 50 μM caused a modest reduction in cell viability, with a viability of 86.4%. These results indicate that **TC-5**, **TC-6**, and **H5** exhibit lower cytotoxicity towards GES-1 cells compared with donepezil.

### PAMPA-BBB evaluation

**Figure 6.**
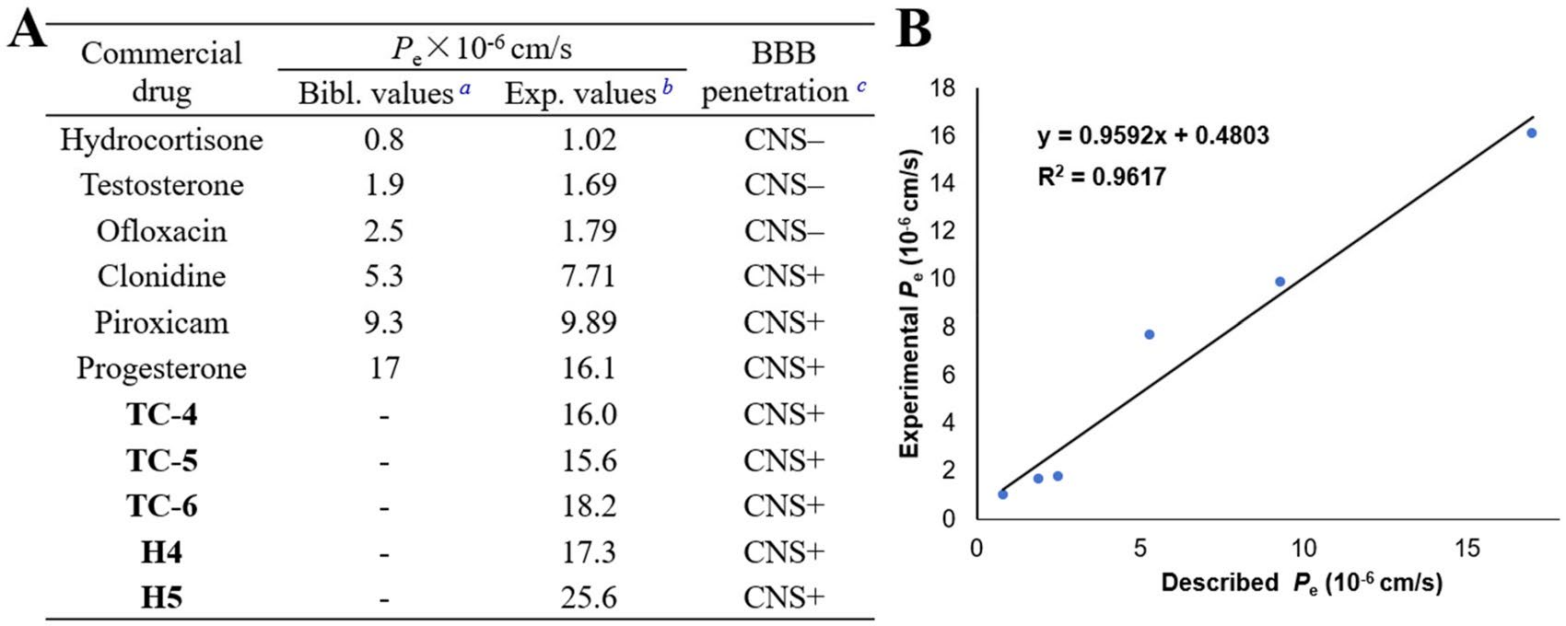
PAMPA-BBB penetration study of **TC-4**, **TC-5**, **TC-6**, **H4** and **H5**. (**A**) Results of the assay. ^a^Bibliographic values represent permeability data reported in the literature; ^b^Results were obtained from three distinct experimental replicates per test; ^c^“CNS +” classification: high (*P*e > 4.0), moderate (2.0–4.0), or low (*P*e < 2.0) permeability, in units of ×10^–6^ cm/s. (**B**) Linear correlation between experimental and literature-derived permeability data for commercial reference drugs, expressed as Pe (experimental) = 0.9592 × Pe (bibliographic) + 0.4803, with an *R*^2^ value of 0.9617. Compounds classified as “CNS+” exhibit Pe values exceeding 4.0 × 10^–6 ^cm/s, indicative of high blood–brain barrier permeability.

The central nervous system (CNS) is the primary target for medications aimed at the treatment of Alzheimer’s disease. To evaluate the ability of compounds **TC-4**, **TC-5**, **TC-6**, **H4**, and **H5** to cross the blood–brain barrier (BBB), the PAMPA-BBB assay was employed.^17^^,^^54^ Six commercially available drugs with reported permeability data were used to validate the experimental procedure, and a strong linear correlation was observed between the experimental and reported values. Compounds with Pe values exceeding 4 × 10^–6 ^cm/s are generally considered capable of crossing the BBB. As shown in Figure 6, the *P*_e_ values of **TC-4**, **TC-5**, **TC-6**, **H4**, and **H5** were 1.60, 1.56, 1.82, 1.73, and 2.56 × 10^–5 ^cm/s, respectively. These results indicate that all five carbamates exhibit good blood–brain barrier permeability.

### Neuroprotective study

**Figure 7.**
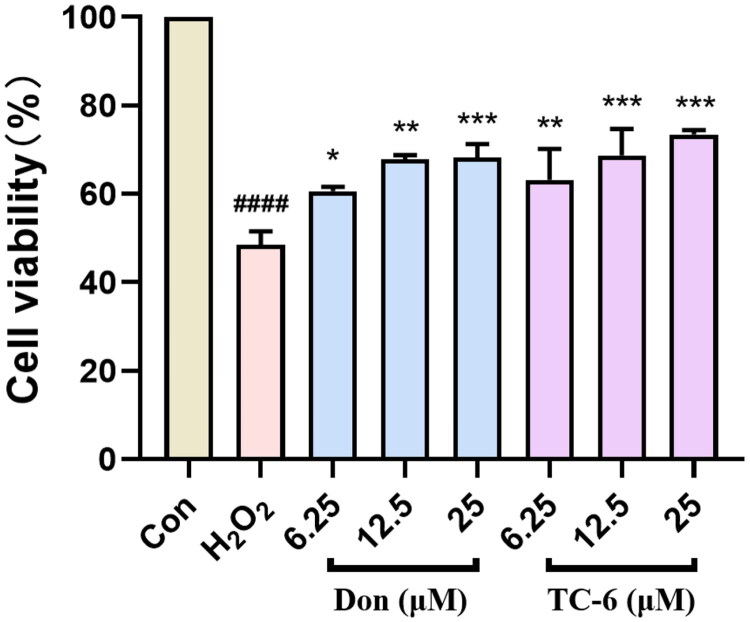
Donepezil and **TC-6** exhibit neuroprotective potential in PC12 cells. Data shown as mean ± SEM (*n* = 3). *p < 0.05, **p < 0.01.***p **<** 0.001 vs. model; ^####^*p* < 0.0001 vs. Control.

The protective effect of compound **TC-6** against H_2_O_2_-induced oxidative damage was evaluated in PC12 cells. Exposure to 100 μM hydrogen peroxide reduced cell viability to 44.7%. As shown in Figure 7, **TC-6** exhibited a concentration-dependent protective effect against H_2_O_2_-induced injury. At a concentration of 25 μM, **TC-6** significantly increased cell viability to 73.0%, comparable to that observed in the donepezil-treated control group. These results suggest that **TC-6** effectively protects PC12 cells from H_2_O_2_-induced oxidative stress, demonstrating notable neuroprotective effects.

### Evaluation of in vivo efficacy of TC-6 in Aβ-induced AD mouse model

The Morris water maze (MWM) is a widely used neurobehavioral test for assessing spatial learning and memory in rodents, including mice and rats. Its maze-based design requires animals to rely on spatial cues to locate a hidden platform beneath the water surface. In Alzheimer’s disease (AD) animal models, intrahippocampal or intracerebroventricular injection of A*β* peptides is commonly used to induce AD-like pathological changes, particularly hippocampal neuronal loss. Behavioural tests were conducted with eight mice per group, for a total of 40 mice included in the study. Two animals died during the surgical procedure and were therefore excluded from the final analysis.

**Figure 8.**
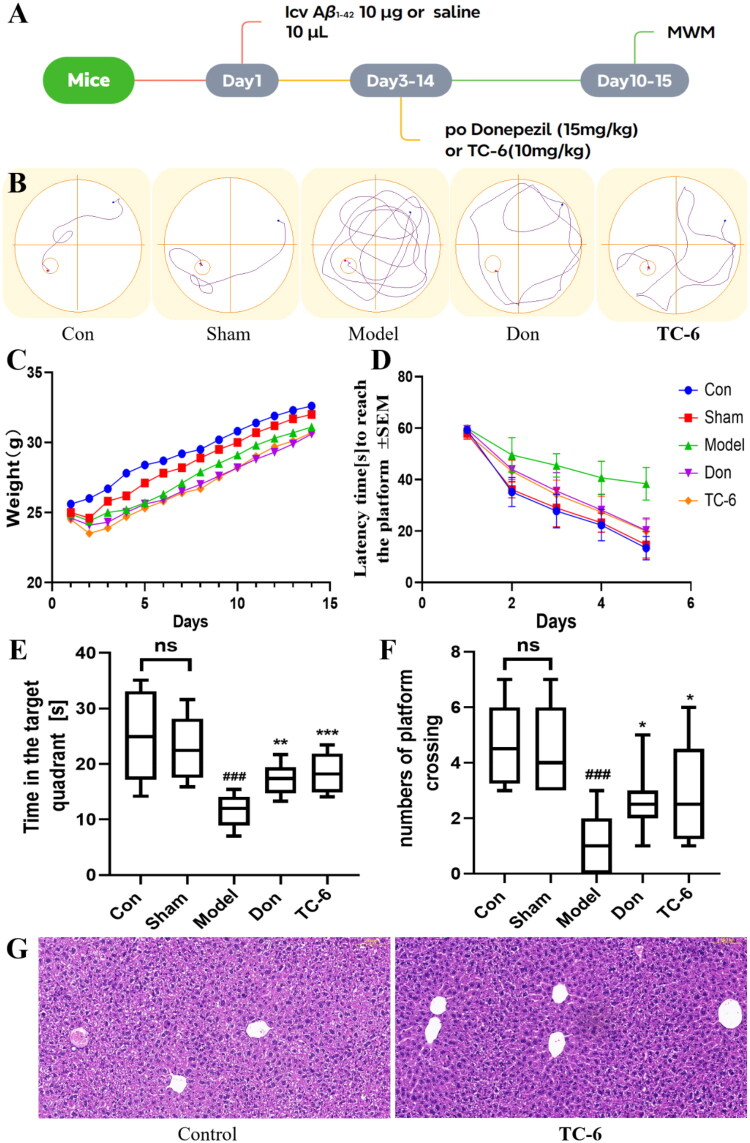
Effects of donepezil and **TC-6** on A*β*_1-42_–induced cognitive impairments in the Morris water maze (MWM) task in 6-week-old male ICR mice. **(A)** Schematic illustration of the in vivo experimental timeline. **(B)** Representative swimming paths of mice in the MWM on the final day of training. **(C)** Daily body weight changes of mice across different treatment groups during the experimental period. **(D)** Learning curves showing escape latency during the acquisition phase for control mice, sham-operated mice receiving intracerebroventricular saline, A*β*_1-42_ model mice (10 μg), and mice treated with **TC-6** (10 mg/kg) or donepezil (15 mg/kg). **(E)** Time spent in the target quadrant during the probe trial. **(F)** Number of platform crossings during the probe trial. **(G)** Representative histological sections of liver from treated and untreated mice. Scale bar = 50 μm. Data are presented as mean ± SD (*n* = 8). ^###^*p* < 0.001 vs control group; *p < 0.05, **p < 0.01, ***p < 0.001 vs A*β*_1-42_ model group.

The cognitive dysfunction model was established by intracerebroventricular injection of A*β*_1-42_, and the effects of compound **TC-6** on cognitive function were evaluated using the Morris water maze (MWM) test. As shown in Figure 8(A), mice received A*β*_1-42_ injection on day 1, followed by drug treatment from days 3 to 14. The five-day MWM training phase was conducted from days 10 to 14 to assess spatial learning and memory acquisition, and a probe trial was performed on day 15 to evaluate memory retention.

As shown in Figure 8(C), body weight changes in all groups remained within the normal range throughout the experiment, indicating good tolerability of both the surgical procedure and the administered compounds. As illustrated in Figure 8(B,D–F), saline-injected mice exhibited no impairment in learning or memory performance. In contrast, mice in the A*β*_1-42_ model group displayed significantly impaired spatial learning and memory compared with the control group. Both the donepezil- and **TC-6**–treated groups showed significant improvements relative to the model group. Notably, donepezil treatment reduced the escape latency, while **TC-6** treatment resulted in even shorter escape latencies, increased platform crossings, and longer time spent in the target quadrant compared with the donepezil group.

**Figure 9.**
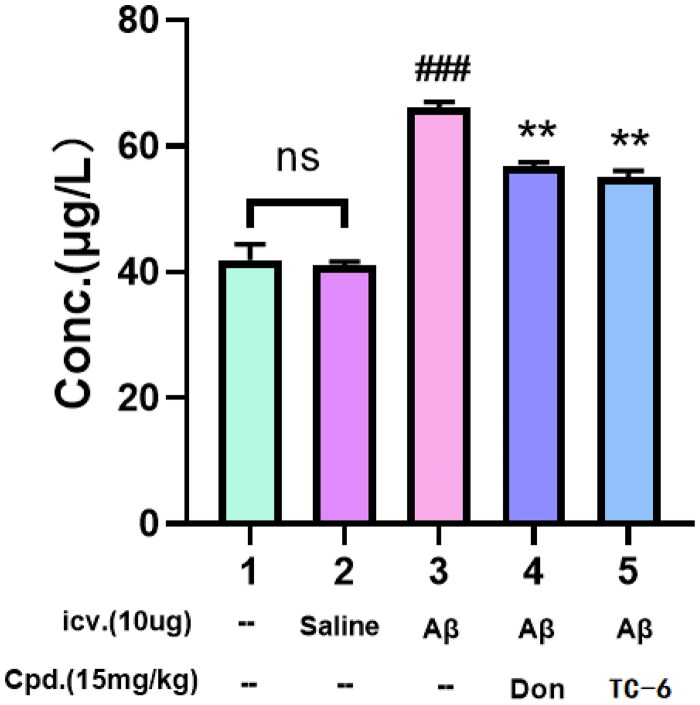
Quantification of total A*β*_1-42_ levels in mouse brains across different groups using an A*β*_1-42_ ELISA kit. ^###^*p* < 0.001 (vs control group), **p < 0.01 vs model group.

As shown in Figure 9, mice in the A*β*_1-42_ group exhibited significantly elevated ventricular A*β* levels compared with the control and sham-operated groups, confirming successful model establishment. Treatment with **TC-6** (10 mg/kg) and donepezil (15 mg/kg) significantly attenuated A*β*_1-42_–induced cognitive impairment, reducing peptide levels by 14.2% and 16.8%, respectively, compared with the model group.

At the end of the behavioural study, mouse livers were collected, fixed in paraffin, and sectioned for histopathological examination. As shown in Figure 8(G), immunohistochemical analysis revealed no evidence of pericentral necrosis or significant steatosis in hepatocytes surrounding the portal vein, indicating the absence of apparent hepatotoxicity.

### Histology examination in hippocampus

The hippocampal CA1 and CA3 regions work in concert to support complex memory functions. The CA3 region is primarily involved in rapid learning and pattern completion, whereas the CA1 region plays a key role in fine memory encoding and retrieval. Dysfunction or damage in either region can result in memory impairment.^17^

To further investigate how the active compound **TC-6** alleviates A*β*_1-42_–induced cognitive deficits, whole mouse brains were harvested for histological examination of the hippocampal CA1 and CA3 regions. Tissue sections were analysed using haematoxylin and eosin (H&E) staining to evaluate the structural integrity of pyramidal neurons. Neuronal density in these hippocampal regions was quantified by manual analysis using ImageJ software. Pyramidal neurons were identified based on their morphology, size, localisation, and the presence of distinct nuclei.

**Figure 10.**
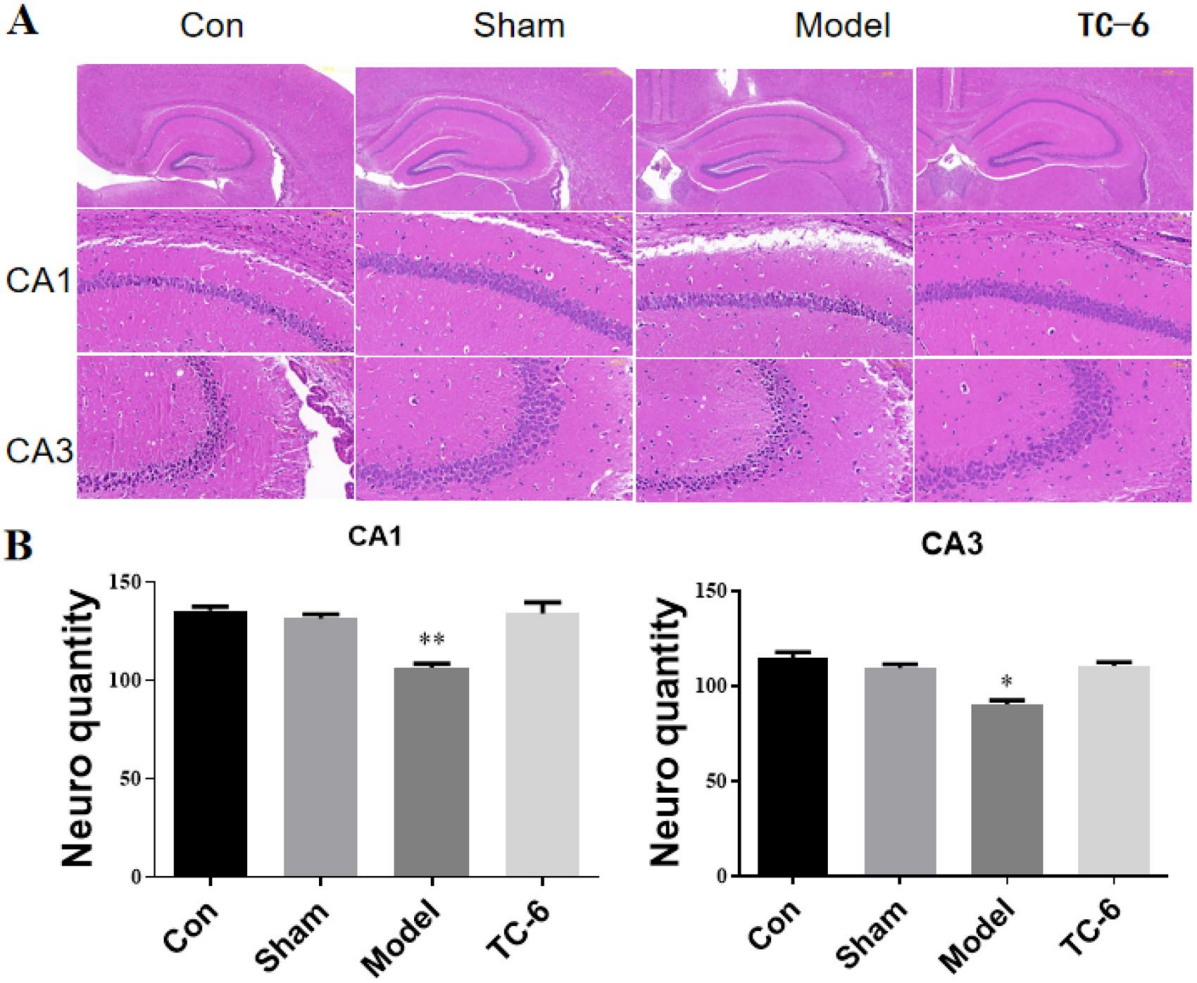
Histological analysis of hippocampal CA1 and CA3 regions. (A) Representative haematoxylin and eosin (H&E)–stained sections of the hippocampus showing the CA1 and CA3 regions. Scale bar = 200 μm (hippocampal overview) and 50 μm (CA1 and CA3 regions); (B) Quantification of neuronal density in the CA1 and CA3 regions. Data are expressed as mean ± SEM (*n* = 3). *p < 0.05, **p < 0.01 vs model group.

As shown in Figure 10(A), A*β*_1-42_–treated mice exhibited a marked reduction in neuronal density, accompanied by a substantial number of degenerating neurons displaying nuclear pyknosis in both the CA1 and CA3 regions. Quantitative analysis (Figure 10(B)) demonstrated a significant increase in neuronal density in the **TC-6**–treated group, particularly in the hippocampal CA1 region.

## Conclusion

Recent studies have identified paeonol-derived carbamate **D12**, originating from a natural phenol, as a dual inhibitor of butyrylcholinesterase (BuChE) and fatty acid amide hydrolase (FAAH). Further analysis revealed that carvacrol carbamates **H4** and **H5** bearing cyclic amines act as selective BuChE inhibitors (IC_50_ = 0.68 and 0.031 μM, respectively), potentially conferring favourable brain exposure, consistent with CBD carbamates. This study established thymol carbamates as a novel class of potent, selective BuChE inhibitors. **TC-6** demonstrated low-nanomolar inhibition of *h*BuChE (IC_50_ = 3.6 nM) with over 2,500-fold selectivity versus *h*AChE.

SAR analysis revealed that: (i) BuChE inhibitory effect of phenolic core (thymol vs. carvacrol), thymol was superior to carvacrol (**TC-5 **>** H4**; **TC-6 **>** H5**); (ii) influence of ring size within cyclic amines, increasing ring size resulted in an initial decrease followed by an enhancement in activity (**TC-4 **>** TC-5 **<** TC-6**; **H4 **<** H5**); and (iii) notably, the low-molecular-weight **TC-6** exhibited the strongest BuChE inhibition. Molecular docking studies confirmed that **TC-6** binds effectively within the hBuChE active site, forming key hydrogen-bond interactions with residues Phe329, Tyr332, Tyr82, and His438 in 1XLU, as well as Arg465 and Phe21 in 4BDS. SAR provided a new framework for the rational design of selective BuChE inhibitors derived from natural phenolic scaffolds. Enzyme kinetic analyses demonstrated that **TC-6** functions as a pseudo-irreversible inhibitor (*k*_d_ = 0.25 μM, *k*_2_ = 0.98 min^−1^), outperforming **H4** and **H5**.

Cytotoxicity and PAMPA-BBB assays indicated favourable safety profiles and blood–brain barrier permeability of **TC-6**. In vivo, **TC-6** significantly alleviated A*β*_1-42_–induced cognitive deficits in mice and reduced hippocampal neurodegeneration. Collectively, these findings identify **TC-6** as a promising therapeutic lead for the treatment of Alzheimer’s disease.

Although the present study provides initial evidence supporting the efficacy and safety of **TC-6**, its safety characterisation remains preliminary. Comprehensive pharmacokinetic, ADME, and toxicological evaluations will be required to fully assess the translational potential of **TC-6** prior to clinical application.

## Experimental section

### Chemistry

#### General information

All reagents were purchased from commercial sources (Energy Chemicals, Shanghai, China) and were used without further purification. Unless noted otherwise, reactions were conducted under ambient air. Reaction progress was monitored by TLC on silica gel plates with UV detection at 254 nm. Melting points were determined using an uncorrected XT4MP apparatus (Taike Instruments, Beijing, China).^1^H and ^13^C NMR spectra were recorded in CDCl_3_ at 500 and 126 MHz, respectively, with chemical shifts (*δ*) in ppm relative to TMS and coupling constants (*J*) in Hz. Signal multiplicities are denoted as *s* (singlet), *d* (doublet), *t* (triplet), and *m* (multiplet). HRMS data were obtained on an Agilent 1260–6221 TOF mass spectrometer. The purity (relative content) of synthesised compounds was determined by HPLC through area normalisation method.

#### General synthesis procedure for carbamates TC-1–TC-12

A solution of diamine (**A**) (3.0 mmol) in 5 mL anhydrous dichloromethane was added dropwise over 1 h to an ice-cooled suspension of 0.84 g sodium bicarbonate and 0.60 g triphosgene (10 mmol and 2.0 mmol, respectively) were added to 20 mL dichloromethane. Once the addition was finished, the mixture was brought to room temperature and stirred for another 6–8 h. After filtration, the resulting filtrate was concentrated under reduced pressure to obtain the crude carbamoyl chloride (B), which was used directly in the subsequent reaction without further purification.

*Synthesis of phenol carbamates* ***TC-1–TC-12***: A mixture of phenol (0.50 mmol), anhydrous K_2_CO_3_ (2.0 equiv), and 4-dimethylaminopyridine (4-DMAP, 0.2 equiv) was dissolved in 5 mL of acetonitrile (MeCN). Carbamoyl chloride B (1.0 or 1.5 equiv) was then added, and the reaction mixture was stirred at 60–65 °C under an argon atmosphere for 5–10 h. TLC analysis was used to follow the reaction progress. Upon completion, evaporation under reduced pressure removed the solvent, and the residue was diluted with water (20 mL) and extracted with dichloromethane (CH_2_Cl_2_, 2 × 30 mL). The combined organic layers were sequentially washed with 5% NaOH (20 mL), 5% HCl (20 mL), and saturated brine (25 mL), then dried over anhydrous Na_2_SO_4_ and filtered. The solvent was removed under vacuum, and the crude product was purified via flash chromatography on silica gel using CH_2_Cl_2_/EtOAc (2: 1) as the eluent to afford the desired compounds **TC-1**–**TC-12**. Characterisation data of **TC-1**–**TC-12** including ^1^H-NMR,^13^C-NMR, and HRMS and their spectra were listed in Supporting Information.

### Assays for EeAChE and eqBuChE enzyme inhibition

Using a modified Ellman’s method, cholinesterase inhibitory activity was determined. Reactions were carried out in 48-well microplates with a total volume of 500 μL per well. The assay buffer consisted of 0.1 M phosphate buffer (pH 8.0), in which either AChE (0.036 U/mL; C3389-500UN, Sigma) or BuChE (0.036 U/mL; C4290-1KU, Sigma) was dissolved. Test compounds at concentrations of 100, 50, 25, 10, and 5 μM were added to different wells, followed by incubation at 37 °C for 20 min. The reaction was initiated by adding 0.35 mM acetylthiocholine iodide (ACh; A5751-1G, Sigma) or 0.5 mM butyrylthiocholine iodide (BuCh; 20820–1 G, Sigma) together with 0.35 mM DTNB (D8130-1G, Sigma). Absorbance was measured at 410 nm after an additional 20 min using a PerkinElmer VICTOR Nivo plate reader.

IC_50_ values were calculated by nonlinear regression analysis using SPSS 24 software. Negative control reactions were performed without inhibitors, and blank wells contained buffer without enzyme or test compounds. Donepezil was used as the positive control. All experiments were independently conducted in triplicate.

### Assays for hAChE and hBuChE enzyme inhibition

The experiment was conducted in 96-well plates with a total amount of 100 μL. Human AChE (C1682, Sigma) and human BuChE (B4186, Sigma) were prepared in 1% gelatine solution and diluted with deionised water to a final activity of 0.125 U/mL. Acetylthiocholine iodide (ACh; A5751-1G, Sigma) and butyrylthiocholine iodide (BuCh; 20820–1 G, Sigma) were prepared in deionised water at a concentration of 3.75 mM. DTNB (5 mM) was dissolved in 0.1 M phosphate buffer (pH 8.0).

Test compounds were dissolved in DMSO and further diluted with ethanol to obtain five concentrations (200, 100, 10, 1, and 0.1 μM). For each well, 40 μL phosphate buffer, 10 μL compound solution, and 10 μL enzyme solution were added, followed by 20 μL DTNB. After incubation at 37 °C for 5 min, the reaction was initiated by adding 20 μL of ACh or BuCh substrate. Absorbance was measured at 412 nm after an additional 5 min at 37 °C.

Blank wells contained buffer instead of enzyme. Enzyme activity was expressed as a percentage of the control and plotted against the logarithm of compound concentration to determine IC_50_ values using SPSS 24 software. All reactions were performed in triplicate in three independent experiments.

### Molecular docking study

The crystal structures of X-ray structures of DFP-inhibited BuChE after ageing (PDB: 1XLU) and hBuChE in complex with tacrine (PDB: 4BDS) were selected as templates. Rivastigmine, a commercially available cholinesterase inhibitor, was utilised for molecular docking in this study. The MOE Protein Align/Superpose settings were utilised to analyse overlap and similarity, focusing on the protein binding pocket. Using Discovery Studio 2019, the protein structure was repaired, partial charges were assigned, and the CHARMM force field was applied to protonate the structure. The ligand was energy-minimized to obtain its lowest-energy conformation. Crystallographic data of the ligand were used to define the protein binding site. CDOCKER simulations were then performed to model ligand–protein interactions and predict binding behaviour. The pose with the lowest CDOCKER_INTERACTION_ENERGY value was considered the most stable and was selected for analysis of binding interactions and visualisation.

### Investigation of inhibition kinetics of eqBuCh

Carbamylation kinetics were measured following the enzyme inhibition assay protocol. Before adding the substrate, the enzyme was pre-incubated with five different concentrations of the inhibitor for 1, 2, 4, 8, 15, 25, 40, and 60 min. The resulting time-dependent inhibition curves were fitted to a first-order exponential decay model to obtain the observed rate constant *k*_obs_ (min^−1^) for each inhibitor concentration.

The dependence of *k*_obs_ on inhibitor concentration was subsequently analysed using double-reciprocal plots, from which the dissociation constant (*k*_d_) and the carbamylation rate constant (*k*_2_) were derived. The decarbamylation rate constant (*k*_3_) was determined by analysing the recovery of enzyme activity following inhibitor removal. Detailed equations and derivations are provided in the Supporting Information.

### Cytotoxicity assays

The GES-1 (human intestinal epithelial cell line, CVCL_EQ22, GNHu43), obtained from Procell Life Science and Technology Co., Ltd. (China), was cultured at 37 °C in a humidified atmosphere containing 5% CO_2_ in DMEM supplemented with 10% foetal bovine serum (FBS; derived from South America; Wisent Inc., Lot No. 086150075). For the MTT assay, cells were seeded at a density of 1 × 10^5^ cells/mL in 96-well plates and incubated for 24 h. The original culture medium was discarded, and fresh medium containing the test compound was added, followed by an additional 24 h incubation. Subsequently, MTT was added in the dark to a final concentration of 5.0 mg/mL, and the plates were incubated for 4–6 h. The medium was then removed, and 150 µL of anhydrous DMSO was added to each well. Absorbance at 492 nm (OD_492_) was measured using a microplate reader. Cell viability (%) was calculated as (OD_492_ of treated cells/OD_492_ of control cells) × 100. Blank wells consisted of cells cultured in fresh medium only, while compound-treated wells consisted of cells treated with the indicated compounds or donepezil. All assays were conducted in triplicate in three independent experiments.

### PAMPA-BBB for blood-brain barrier permeability evaluation

The ability of compounds to penetrate the blood–brain barrier (BBB) was assessed using the PAMPA-BBB assay, based on the protocol developed by Di et al.^54^ The system was evaluated and validated using six commercially available drugs purchased from Aladdin Reagents. DMSO and dodecane were obtained from Energy Chemical. The experimental setup consisted of a 96-well donor plate with 0.45 μm PVDF membranes (MAIPN455, Millipore) and a matching receiver plate (MATRNPS50, Millipore), along with a UV-transparent 96-well plate (REF-3635, CO-STAR, Corning). Stock solutions of test and reference compounds (20 mg/mL in DMSO) were diluted 1:200 with phosphate-buffered saline (PBS, pH 7.4 ± 0.1) and ethanol in a 70:30 (v/v) ratio to obtain a final concentration of 100 μg/mL. Prior to assembly, 4 μL of porcine brain lipid (PBL, 20 mg/mL in dodecane; 141101 P-100 mg, Avanti Polar Lipids) was applied to the donor membrane. Subsequently, the test solution (200 μL) was added to each donor well, while 300 μL of PBS–ethanol mixture was added to the corresponding receiver wells. The donor plate was then placed onto the receiver plate to form a sandwich configuration, ensuring membrane–buffer contact. The assembled plates were incubated at 25 °C for 18 h. After incubation, compound concentrations in both donor and receiver wells were quantified using a PerkinElmer VICTOR Nivo UV spectrophotometer. Measurements were performed at three wavelengths, with at least four replicates per condition across three independent experiments.

### Neuroprotective study

PC12 (rat adrenal pheochromocytoma cells, CVCL-0481, ATCC CRL-1721), obtained from Procell Life Science and Technology Co., Ltd. (China), were seeded into 96-well plates at a density of 10,000 cells per well and allowed to adhere overnight. On the following day, cells were treated with **TC-6** at concentrations of 6.25, 12.5, and 25 μM for 3 h. Subsequently, hydrogen peroxide (100 μM) was added to induce oxidative stress, followed by an additional 24 h incubation in fresh medium containing the same concentration of **TC-6**. Cell viability was assessed using the MTT assay as described above.

### Animal studies

ICR mice (6–8 weeks old, weighing 18–24 g, male) were purchased from the Animal Centre of Anhui Medical University (Hefei, China). All procedures were conducted in accordance with the National Institutes of Health Guide for the Care and Use of Laboratory Animals. Animal housing and experimental protocols were approved by the Institutional Animal Care and Use Committee of Anhui Medical University (Approval No. LLSC20240861, March 1, 2024). All experiments complied with the ARRIVE guidelines. Under SPF-controlled conditions (22 ± 2 °C, 12 h light/dark cycle, 55 ± 5% humidity), animals were provided *ad libitum* access to commercial solid diet and water for at least one week prior to experimentation.

Upon completion of in vivo procedures, mice were humanely euthanized by cervical dislocation. Forty male ICR mice were randomly assigned to five groups (*n* = 8 per group): (1) solvent group (blank control, ig); (2) sham group receiving intracerebroventricular injection of normal saline; (3) A*β*_1-42_ oligomer group receiving intracerebroventricular administration of oligo-A*β*_1-42_ peptide (10 μg); (4) positive control group receiving oligo-A*β*_1-42_ peptide (10 μg, icv) and donepezil (15 mg/kg, oral); and (5) treatment group receiving oligo-A*β*_1-42_ peptide (10 μg, icv per mouse) and **TC-6** (10 mg/kg, gavage). Prior to administration, both the reference drug and **TC-6** were prepared as suspensions in a DMSO–saline solution (1:99, v/v).

The Morris water maze (MWM) apparatus consisted of a circular gray tank (1.20 m diameter, 0.60 m height) with adjustable elevation and positioning. Using SMART software (version 3.0, Panlab, Spain), the pool was divided into four equal quadrants: northeast, northwest, southeast, and southwest. The maze was illuminated at 45 lx during testing. Animals relied on spatial cues around the pool to locate the hidden platform. The experiment consisted of a training phase and a test phase. During the training phase, mice were placed in the pool and required to locate the submerged platform. Training lasted for 5 consecutive days, with four trials per day from different starting positions (east, west, south, or north), randomly assigned. If an animal failed to locate the platform within 90 s, it was guided to the platform. On day 6, the probe test was conducted with the platform removed. Mice were allowed to swim freely for 60 s to search for the former platform location; if the platform area was not crossed within this time, the latency was recorded as 60 s. Performance was evaluated based on swimming trajectory, escape latency, and the number of crossings over the former platform location.

In the A*β*_1-42_ oligomer–induced injury study, mice were euthanized after behavioural testing, and brain tissues were collected for analysis. Total A*β*_1-42_ levels were quantified using a commercial mouse ELISA kit (CSB-E10787m, Wuhan Yipu Biotechnology Co., Ltd., China). A*β*_1-42_ concentrations were calculated from a standard curve generated by linear regression. Data were analysed using GraphPad Prism 8.0 and are presented as mean ± SEM.

### Histology examination in hippocampus

Brain tissue samples were collected from three mice per experimental group and preserved in a 4% paraformaldehyde solution. This fixation process served two critical purposes: preventing post-mortem cellular breakdown (autolysis) and inhibiting bacterial degradation, thereby maintaining the tissue’s original cellular architecture. Following paraffin embedding, researchers examined neuronal morphology and pathological alterations specifically within the hippocampal CA1 and CA3 subregions.

### Statistical analysis

Mean ± SEM from a minimum of three separate studies; analysed using GraphPad Prism 8.

## Supplementary Material

Supporting Information_ Clean.docx

## Data Availability

The authors confirm that the data supporting the findings of this study are available within the article and/or its supplementary materials.
