# Supplementary material for: Thymol carbamates bearing cyclic amines as potent and selective BuChE inhibitors alleviate memory impairments for Alzheimer’s disease therapy
Source: J Enzyme Inhib Med Chem. 2026 Feb 6;41(1):2623314. doi: 10.1080/14756366.2026.2623314 (PMC12885020; doi:10.1080/14756366.2026.2623314)
Supplement: Supporting Information_ Clean.docx [file IENZ_A_2623314_SM3424.docx]

**Supporting Information**

**Thymol carbamates bearing cyclic amines as potent and selective BuChE inhibitors** **alleviate memory impairments for Alzheimer’s disease therapy**

Chengyao Wu ^a,1^, Yulu Ding ^b,1^, Xiaodan Liu ^b^, Shan Gao ^b^, Xiaoqing Wang ^a,*^, Wenjian Tang ^b,*^

*^a^ The First People's Hospital of Chuzhou, The Affiliated Chuzhou Hospital of Anhui Medical University, Chuzhou239001, China.*

*^b^ School of Pharmacy,* *Anhui Medical University, Hefei 230032, China*

**Table of contents**

1. Characterization data of **TC-1–TC-12**....................................................................S2

2. **Table S1**. Information of the HPLC........................................................................S6

3. The copies of NMR, HRMS (ESI) and HPLC spectra of **TC-1–TC-12**.................S7

*2-isopropyl-5-methylphenyl methylcarbamate (****TC-1****).* White crystal, yield 77%, purity 95.9%, m.p.: 87.5‒90.0℃; ^1^H NMR (600 MHz, CDCl_3_) *δ* 7.24 (d, *J* = 7.9 Hz, 1H), 7.11 (d, *J* = 7.8 Hz, 1H), 7.04 (s, 1H), 5.15 (s, 1H), 3.11 – 3.07 (m, 1H), 3.00 (q, *J* = 6.4, 5.1 Hz, 3H, NCH_3_), 2.29 (s, 3H, CH_3_), 1.40 – 1.32 (m, 6H, CH*Me_2_*); ^13^C NMR (151 MHz, CDCl_3_) *δ* 155.34 (C=O), 149.48, 148.00, 130.88, 127.79, 123.81, 120.24, 33.67, 27.87, 24.03 (2C, CH*Me_2_*), 15.79; TOF-HRMS (ESI) *m/z*: [M + H]^+^, calcd for C_12_H_18_NO_2_: 208.1332; found: 208.1330.

*2-isopropyl-5-methylphenyl dimethylcarbamate (****TC-2****).* Colorless oil, yield 81%, purity 95.5%; ^1^H NMR (600 MHz, CDCl_3_) *δ* 7.18 (d, *J* = 7.9 Hz, 1H), 7.00 (dd, *J* = 8.0, 1.8 Hz, 1H), 6.93 – 6.83 (m, 1H), 3.13 (s, 3H, NCH_3_), 3.09 – 3.05 (m, 1H), 3.03 (s, 3H, NCH_3_), 2.32 (s, 3H, CH_3_), 1.23 (d, *J* = 7.0 Hz, 6H, CH*Me_2_*); ^13^C NMR (151 MHz, CDCl_3_) *δ* 155.16 (C=O), 148.71, 137.25, 136.40, 126.54, 126.16, 123.21, 36.82, 36.47, 27.23, 23.09 (2C, CH*Me_2_*), 20.91; TOF-HRMS (ESI) *m/z*: [M + H]^+^, calcd for C_13_H_20_NO_2_: 222.1489; found: 222.1487.

*2-isopropyl-5-methylphenyl ethyl(methyl)carbamate (****TC-3****).* Colorless oil, yield 76%, purity 99.5%; ^1^H NMR (400 MHz, CDCl_3_) *δ* 7.16 (d, *J* = 7.9 Hz, 1H), 6.98 (dd, *J* = 8.1, 1.8 Hz, 1H), 6.88 (d, *J* = 7.9 Hz, 1H), 3.55 – 3.37 (m, 2H), 3.05 (d, *J* = 34.8 Hz, 4H), 2.31 (d, *J* = 0.7 Hz, 3H, CH_3_), 1.29 – 1.23 (m, 1H), 1.21 (d, *J* = 7.0 Hz, 6H, CH*Me_2_*); ^13^C NMR (126 MHz, CDCl_3_) *δ* 154.92 (C=O), 148.74, 137.34, 136.48, 126.56, 126.17, 123.16, 44.18, 34.40, 27.20, 23.11 (2C, CH*Me_2_*), 20.96, 13.38. TOF-HRMS (ESI) *m/z*: [M + H]^+^, calcd for C_14_H_22_NO_2_: 236.1645; found: 236.1646.

*2-isopropyl-5-methylphenyl azetidine-1-carboxylate (****TC-4****).* Colorless oil, yield 79%, purity 99.1%; ^1^H NMR (500 MHz, CDCl_3_) *δ* 7.16 (d, *J* = 7.9 Hz, 1H), 7.02 – 6.96 (m, 1H), 6.93 – 6.87 (m, 1H), 4.18 (m, 4H), 3.06 (p, *J* = 6.9 Hz, 1H), 2.37 – 2.32 (m, 2H), 2.31 (s, 3H, CH_3_), 1.21 (d, *J* = 6.9 Hz, 6H, CH*Me_2_*); ^13^C NMR (126 MHz, CDCl_3_) *δ* 154.73 (C=O), 148.25, 137.35, 136.47, 126.64, 126.28, 123.13, 50.34, 49.33, 27.25, 23.08 (2C, CH*Me_2_*), 20.96, 15.88; TOF-HRMS (ESI) *m/z*: [M + H]^+^, calcd for C_14_H_20_NO_2_: 234.1489; found: 234.1489.

*2-isopropyl-5-methylphenyl pyrrolidine-1-carboxylate (****TC-5****).* Colorless oil, yield 80%, purity 97.6%; ^1^H NMR (600 MHz, CDCl_3_) *δ* 7.16 (d, *J* = 7.9 Hz, 1H), 6.97 (dd, *J* = 7.9, 1.8 Hz, 1H), 6.92 (d, *J* = 1.8 Hz, 1H), 3.58 (t, *J* = 6.7 Hz, 2H), 3.49 (t, *J* = 6.7 Hz, 2H), 3.13 – 3.03 (m, 1H), 2.30 (s, 3H, CH_3_), 2.01 – 1.88 (m, 4H), 1.21 (d, *J* = 6.9 Hz, 6H, CH*Me_2_*); ^13^C NMR (151 MHz, CDCl_3_) *δ* 153.45 (C=O), 148.62, 137.23, 136.35, 126.43, 126.14, 123.24, 46.53, 46.39, 27.29, 25.94, 25.10, 23.07 (2C, CH*Me_2_*), 20.95; TOF-HRMS (ESI) *m/z*: [M + H]^+^, calcd for C_15_H_22_NO_2_: 248.1645; found: 248.1646.

*2-isopropyl-5-methylphenyl piperidine-1-carboxylate (****TC-6****).* Colorless oil, yield 78%, purity 98.9%;^1^H NMR (400 MHz, CDCl_3_) *δ* 7.16 (d, *J* = 7.9 Hz, 1H), 6.98 (dd, *J* = 8.0, 1.8 Hz, 1H), 6.88 (d, *J* = 1.8 Hz, 1H), 3.58 (d, *J* = 43.1 Hz, 4H), 3.04 (p, *J* = 6.9 Hz, 1H), 2.31 (s, 3H, CH_3_), 1.71 – 1.59 (m, 6H), 1.21 (d, *J* = 6.9 Hz, 6H, CH*Me_2_*); ^13^C NMR (101 MHz, CDCl_3_) *δ* 154.02 (C=O), 148.78, 137.30, 136.45, 126.52, 126.19, 123.33, 45.65, 45.29, 27.34, 26.20, 25.76, 24.51, 23.10 (2C, CH*Me_2_*), 20.96. TOF-HRMS (ESI) *m/z*: [M + H]^+^, calcd for C_16_H_24_NO_2_: 262.1802; found: 262.1803.

*2-isopropyl-5-methylphenyl benzyl(methyl)carbamate (****TC-7****).* Colorless oil, yield 75%, purity 98.5%; ^1^H NMR (400 MHz, CDCl_3_) *δ* 7.42 – 7.28 (m, 5H), 7.18 (t, J = 7.3 Hz, 1H), 7.00 (d, J = 7.9 Hz, 1H), 6.91 (d, J = 23.0 Hz, 1H), 4.63 (d, J = 42.3 Hz, 2H), 3.13 – 2.93 (m, 4H), 2.32 (d, J = 5.2 Hz, 3H), 1.19 (dd, J = 28.2, 6.9 Hz, 6H, CH*Me_2_*).TOF-HRMS (ESI) *m/z*: [M + H]^+^, calcd for C_19_H_24_NO_2_: 298.1802; found:298.1805.

*2-isopropyl-5-methylphenyl (3-chlorobenzyl)(methyl)carbamate (****TC-8****).* Colorless oil, yield 77%, purity 96.6%; ^1^H NMR (400 MHz, DMSO-*d*_6_) *δ* 7.14 (dt, *J* = 24.4, 9.3 Hz, 3H), 7.03 (dd, *J* = 12.0, 7.5 Hz, 1H), 6.93 (dd, *J* = 18.6, 7.9 Hz, 1H), 6.75 (t, *J* = 9.5 Hz, 1H), 6.59 (d, *J* = 32.4 Hz, 1H), 4.33 (d, *J* = 60.1 Hz, 2H), 2.75 (d, *J* = 25.2 Hz, 4H), 2.00 (d, *J* = 8.0 Hz, 3H, CH_3_), 1.12 – 0.40 (m, 6H, CH*Me_2_*); ^13^C NMR (126 MHz, CDCl_3_) *δ* 155.59 (C=O), 155.03, 148.60, 139.54, 137.42, 136.60, 134.87, 130.15, 127.91, 127.31, 126.87, 126.25, 125.36, 52.57, 35.27, 27.38, 23.15 (2C, CH*Me_2_*), 20.98. TOF-HRMS (ESI) *m/z*: [M + H]^+^, calcd for C_19_H_23_ClNO_2_: 332.1412; found: 332.1403.

*2-isopropyl-5-methylphenyl (3-bromobenzyl)(methyl)carbamate (****TC-9****).* Colorless oil, yield 76%, purity 99.2%; ^1^H NMR (400 MHz, DMSO-*d*_6_) *δ* 7.61 – 7.43 (m, 2H), 7.35 (dd, *J* = 12.4, 9.3 Hz, 2H), 7.18 (dd, *J* = 18.3, 7.8 Hz, 1H), 7.00 (dd, *J* = 10.6, 7.8 Hz, 1H), 6.84 (d, *J* = 31.5 Hz, 1H), 4.65 (s, 1H), 4.50 (s, 1H), 3.00 (d, *J* = 25.5 Hz, 3H, NCH_3_), 2.26 (d, *J* = 7.6 Hz, 3H, CH_3_), 1.15 (d, *J* = 6.9 Hz, 3H, CH_3_), 1.02 (d, *J* = 6.9 Hz, 3H, CH_3_); ^13^C NMR (101 MHz, DMSO) *δ* 154.68 (C=O), 148.30, 140.75, 136.99, 135.95, 130.86, 130.26, 129.49, 126.48, 126.13, 125.95, 123.23, 122.00, 51.57, 35.28, 26.75, 22.83 (2C, CH*Me_2_*), 20.34; TOF-HRMS (ESI) *m/z*: [M + H]^+^, calcd for C_19_H_23_BrNO_2_: 376.0907; found: 376.0910.

*2-isopropyl-5-methylphenyl (4-chlorobenzyl)(methyl)carbamate (****TC-10****).* Colorless oil, yield 74%, purity 98.3%; ^1^H NMR (500 MHz, CDCl_3_) *δ* 7.36 (s, 2H), 7.28 – 7.24 (m, 2H), 7.17 (t, *J* = 7.5 Hz, 1H), 7.00 (d, *J* = 7.9 Hz, 1H), 6.88 (d, *J* = 31.2 Hz, 1H), 4.58 (d, *J* = 55.4 Hz, 2H), 3.08 – 2.91 (m, 4H), 2.31 (d, *J* = 5.2 Hz, 3H, CH_3_), 1.23 – 1.13 (m, 6H, CH*Me_2_*); ^13^C NMR (101 MHz, CDCl_3_) *δ* 154.12 (C=O), 152.98, 148.73, 137.29, 136.50, 131.50, 126.57, 126.20, 123.32, 121.31, 116.07, 27.35, 26.72, 24.49, 23.10 (2C, CH*Me_2_*), 22.79, 20.94. TOF-HRMS (ESI) *m/z*: [M + H]^+^, calcd for C_19_H_23_ClNO_2_: 332.1412; found: 332.1416.

*2-isopropyl-5-methylphenyl 3,4-dihydroisoquinoline-2(1H)-carboxylate (****TC11****).* white crystal, yield 74%, purity 99.5%, m.p.: 49.5~51.8℃; ^1^H NMR (600 MHz, CDCl_3_) *δ* 7.33 (dd, *J* = 18.8, 8.4 Hz, 1H), 7.18 (t, *J* = 6.0 Hz, 1H), 7.07 (t, *J* = 6.7 Hz, 1H), 6.89 (t, *J* = 7.5 Hz, 1H), 6.81 (dd, *J* = 20.5, 8.4 Hz, 1H), 6.70 (s, 1H), 6.60 (s, 1H), 5.17 (dd, *J* = 9.0, 3.7 Hz, 2H), 3.70 – 3.42 (m, 1H), 3.03 (s, 2H), 2.94 (s, 2H), 2.18 (d, *J* = 15.2 Hz, 3H, CH_3_), 1.11 (d, *J* = 7.3 Hz, 6H, CH*Me_2_*); ^13^C NMR (151 MHz, CDCl_3_) *δ* 160.42 (C=O), 154.96, 148.52, 140.64, 137.05, 136.44, 129.06, 128.16, 126.85, 126.61, 125.79, 123.20, 115.86, 46.71, 37.52, 35.30, 27.37, 23.14 (2C, CH*Me_2_*), 20.86; TOF-HRMS (ESI) *m/z*: [M + H]^+^, calcd for C_20_H_24_NO_2_: 310.1802; found: 310.1795.

1. *isopropyl-5-methylphenyl*

*6,7-dimethoxy-3,4-dihydroisoquinoline-2(1H)-carboxylate (****TC-12****).* White powder, yield 84%, purity 99.1%, m.p.: 111.5~112.4℃; ^1^H NMR (400 MHz, DMSO-*d*_6_) *δ* 7.20 (d, *J* = 7.8 Hz, 1H), 7.01 (d, *J* = 7.9 Hz, 1H), 6.90 – 6.73 (m, 3H), 4.63 (d, *J* = 75.2 Hz, 2H), 3.73 (d, *J* = 8.1 Hz, 6H), 3.36 (s, 1H), 2.81 (dt, *J* = 26.8, 5.7 Hz, 2H), 2.26 (s, 3H, CH_3_), 1.13 (dd, *J* = 11.3, 6.7 Hz, 6H, CH*Me_2_*); ^13^C NMR (126 MHz, CDCl_3_) *δ* 154.29 (C=O), 154.02, 148.49, 147.80, 137.24, 136.48, 126.69, 126.43, 126.21, 126.04, 123.16, 111.57, 109.17, 55.99, 45.74, 42.41, 28.73, 27.24, 23.07 (2C, CH*Me_2_*), 20.88. TOF-HRMS (ESI) *m/z*: [M + H]^+^, calcd for C_22_H_28_NO_4_:370.2013; found: 370.2014.

**Table S1**. Information of the HPLC

| **Compd.** | **liquidity ratio** **(MeCN: H_2_O)** | **proportion %** |
| --- | --- | --- |
| **TC-1** | 1:1 | 95.908 |
| **TC-2** | 1:1 | 95.478 |
| **TC-3** | 1:1 | 99.555 |
| **TC-4** | 1:1 | 99.119 |
| **TC-5** | 7:3 | 97.681 |
| **TC-6** | 7:3 | 98.965 |
| **TC-7** | 8:2 | 98.485 |
| **TC-8** | 1:1 | 96.554 |
| **TC-9** | 8:2 | 99.193 |
| **TC-10** | 8:2 | 98.349 |
| **TC-11** | 8:2 | 99.467 |
| **TC-12** | 8:2 | 99.155 |

***
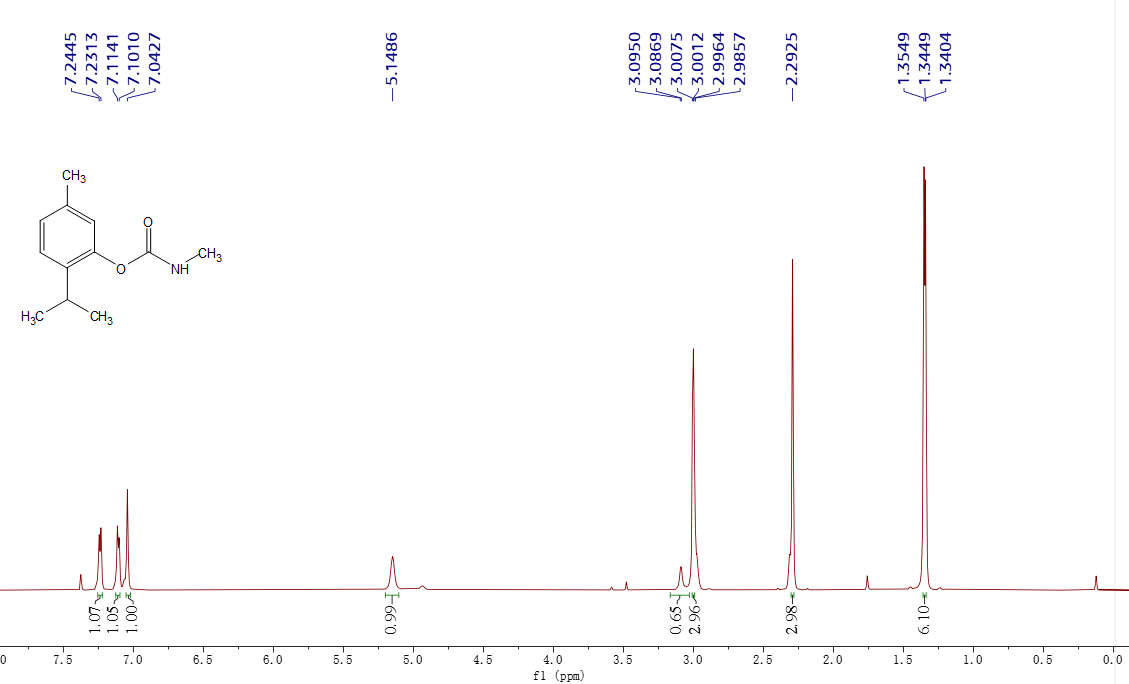
***

***
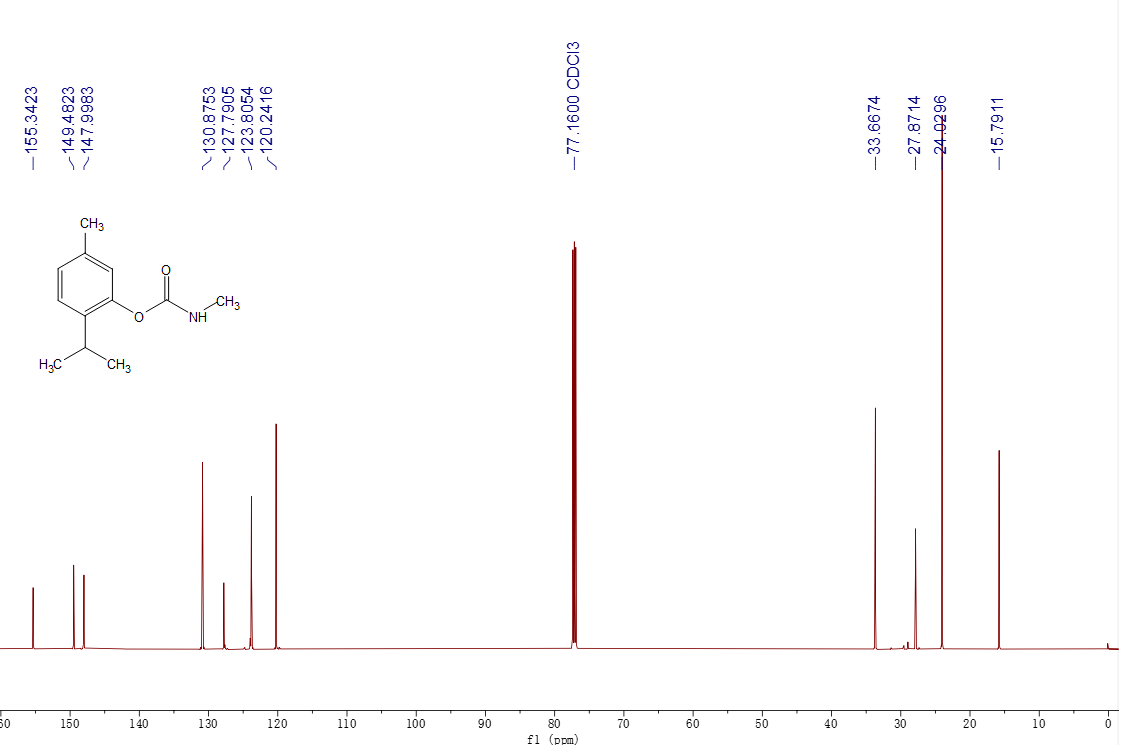
***
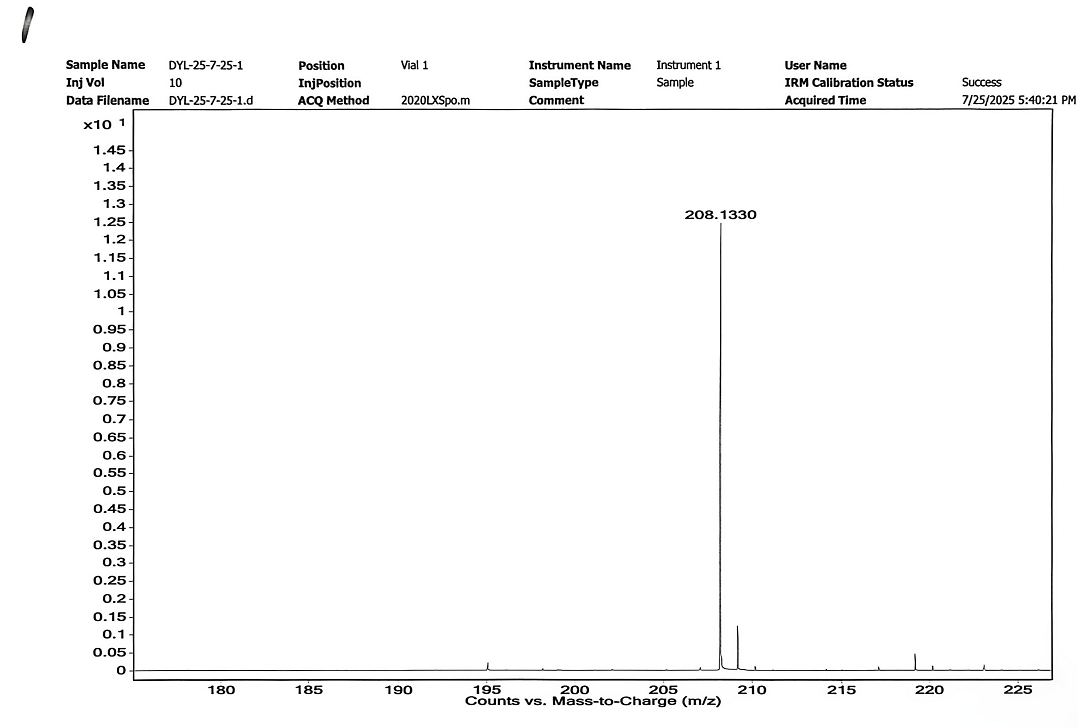

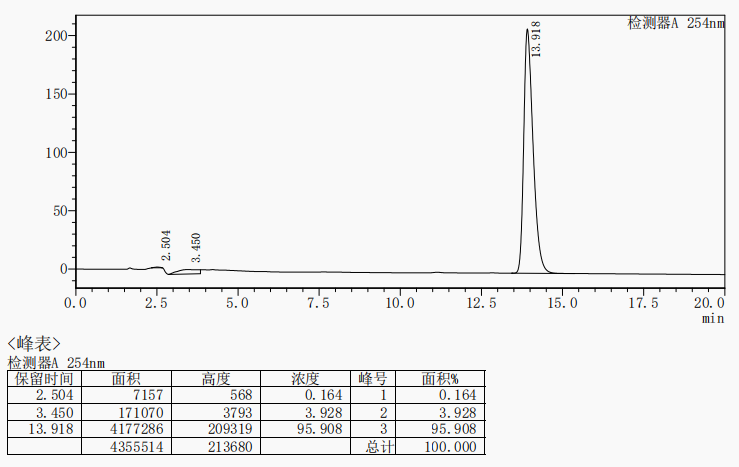
**Figure S1.** ^1^H-NMR, ^13^C-NMR, HRMS (ESI) and HPLC spectra of compound **TC-1.**

***
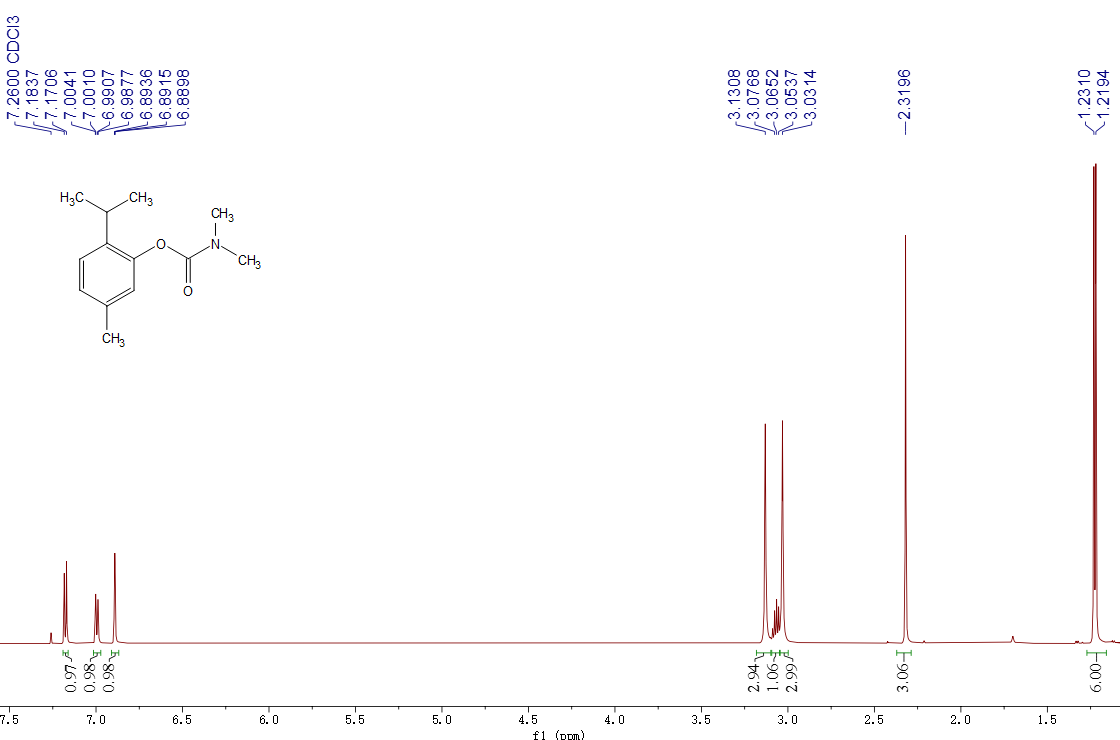
***

***
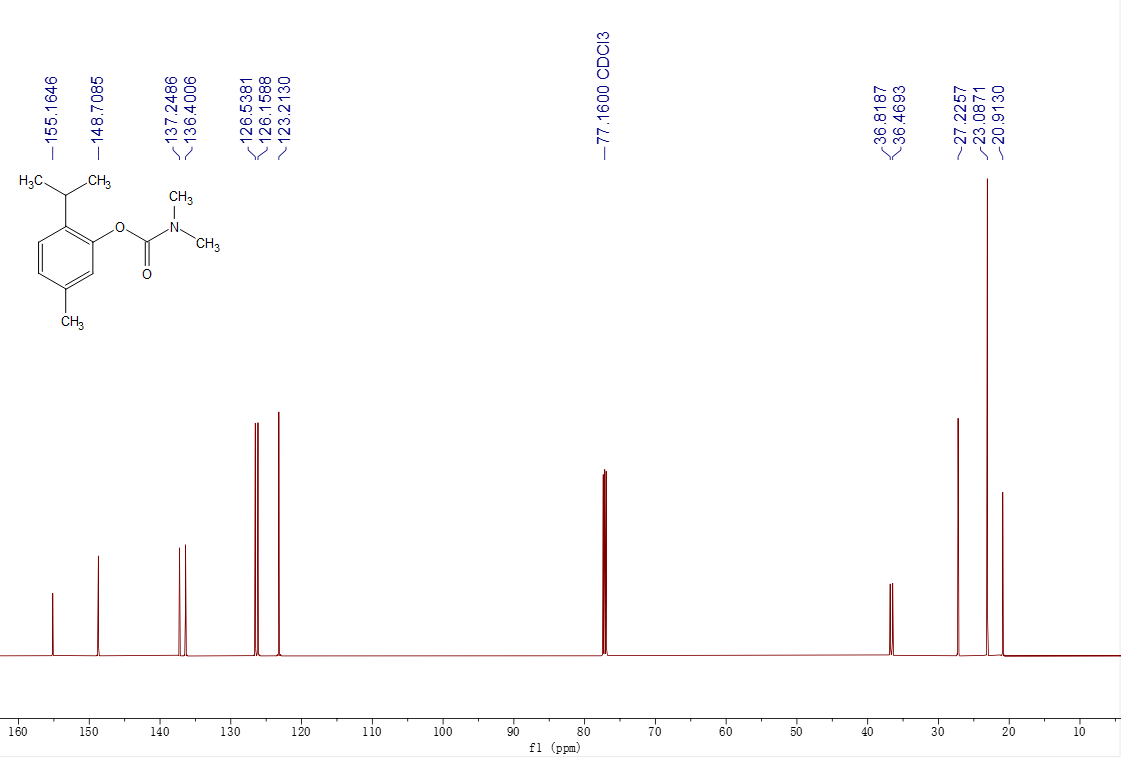
*
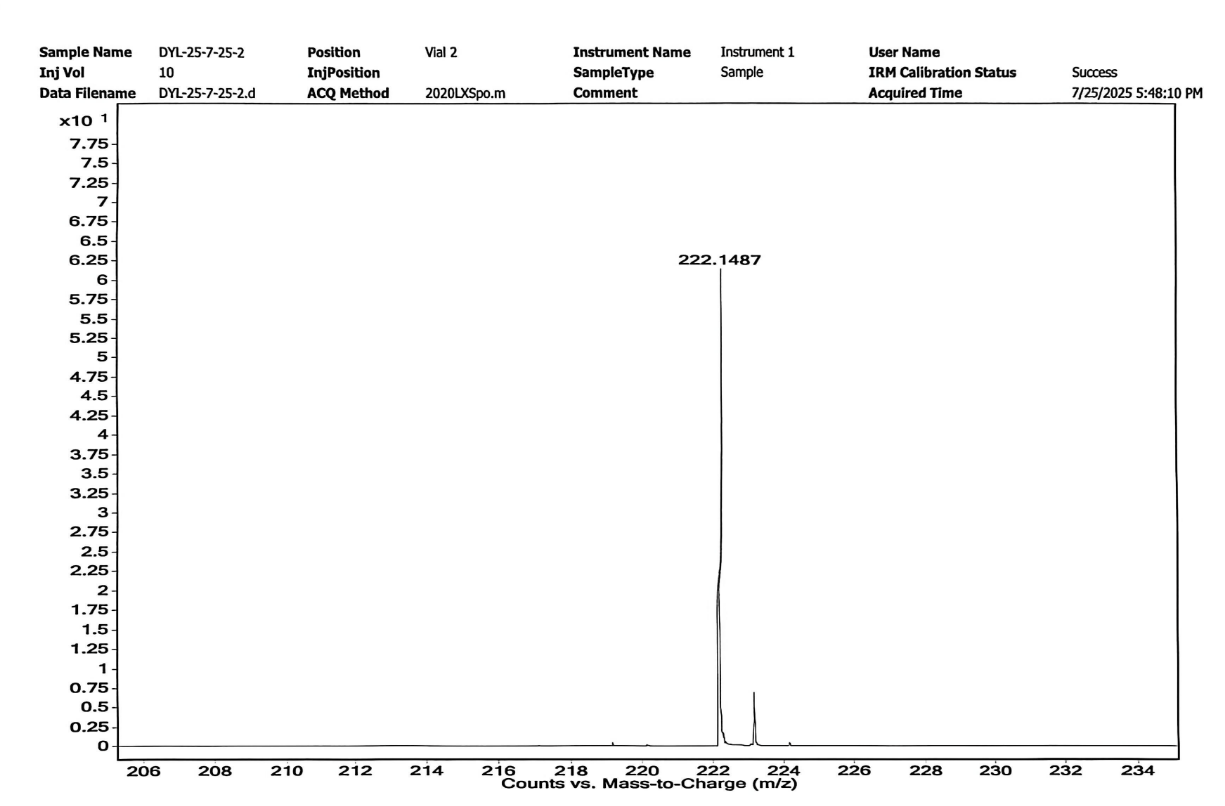
**
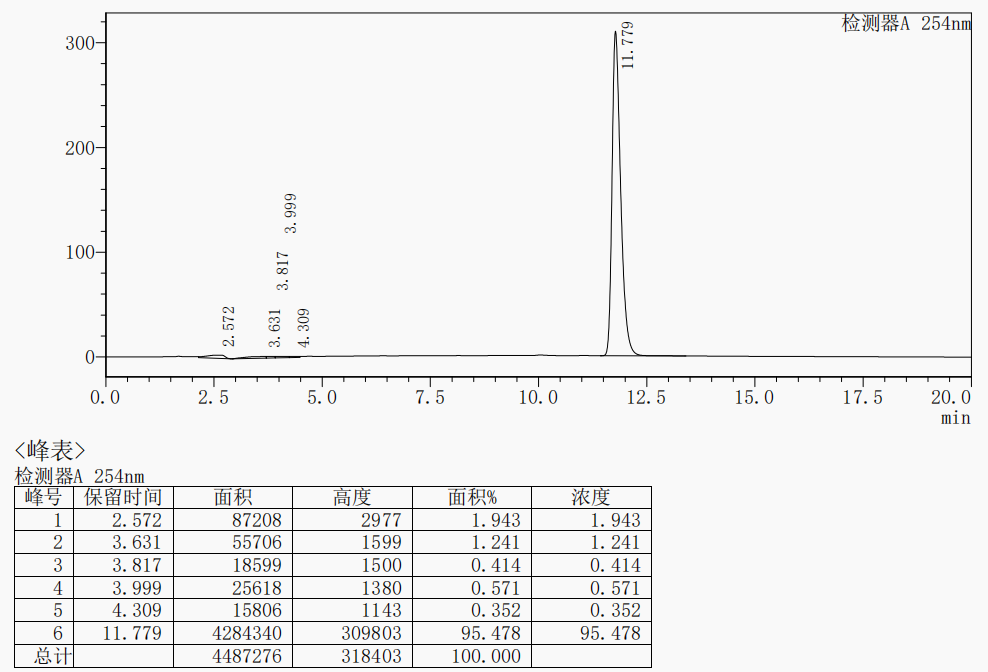
**Figure S2.** ^1^H-NMR, ^13^C-NMR, HRMS (ESI) and HPLC spectra of compound **TC-2.**

***
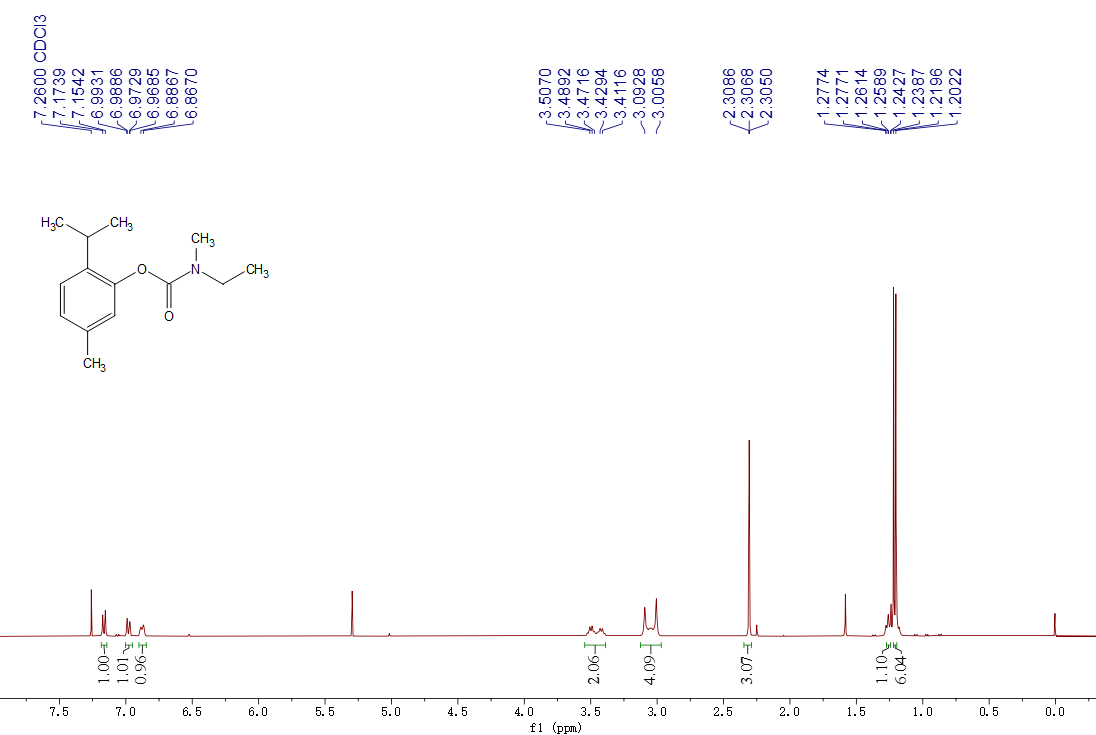
*** ***
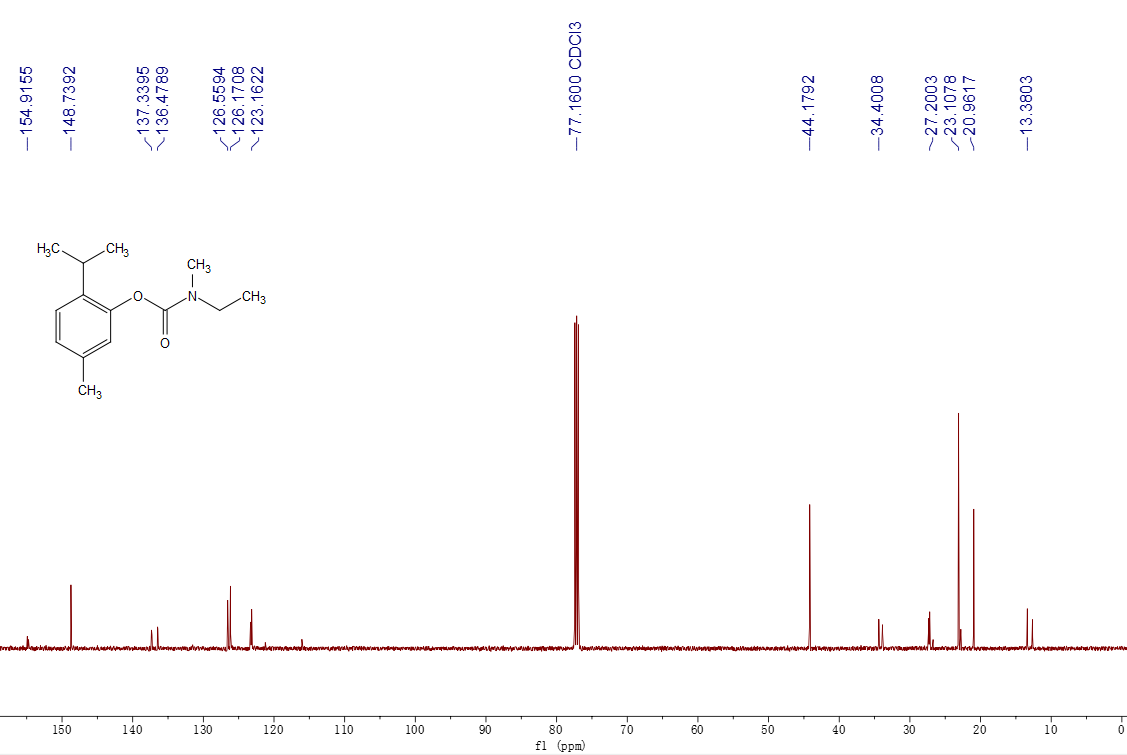
***
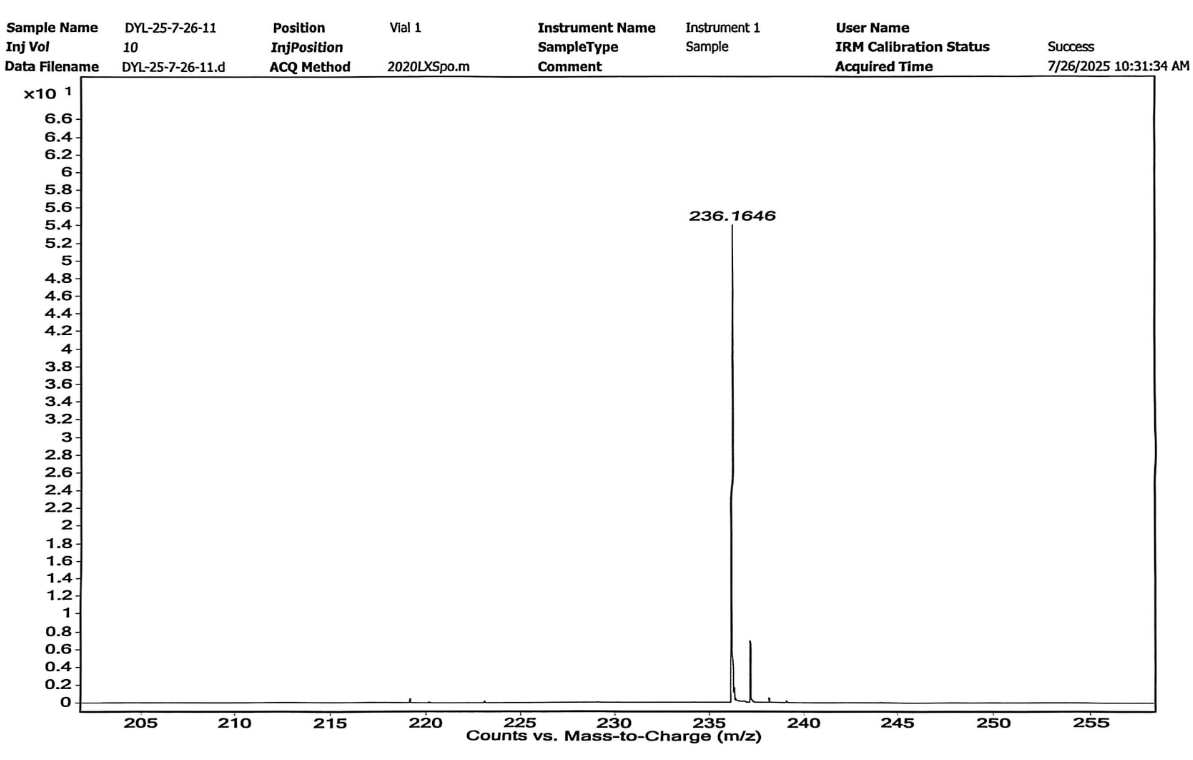

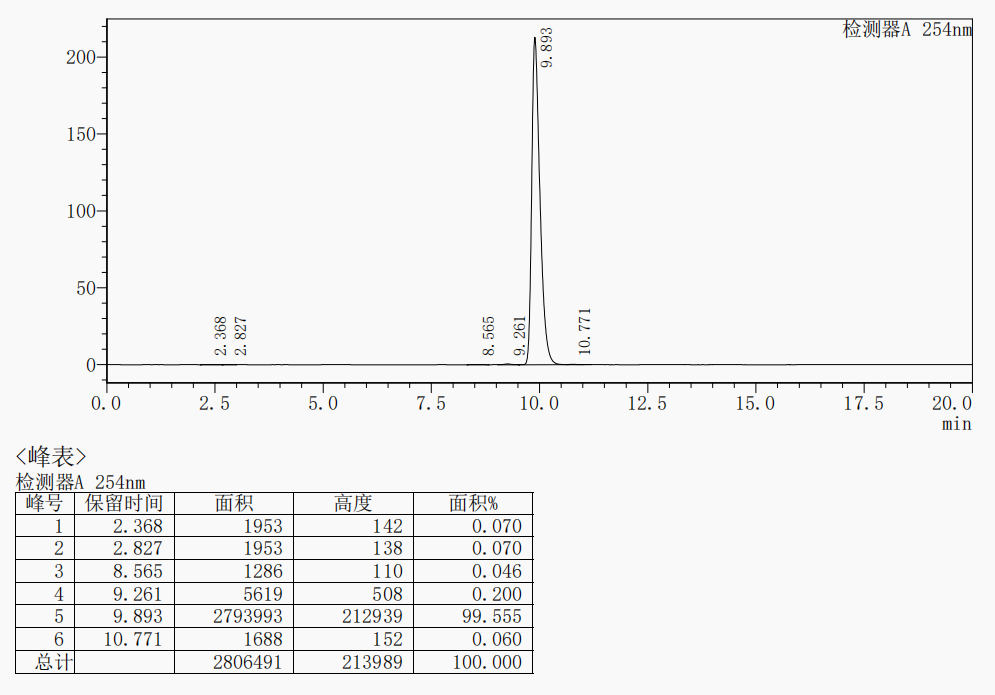


**Figure S3.** ^1^H-NMR, ^13^C-NMR, HRMS (ESI) and HPLC spectra of compound **TC-3.**

***
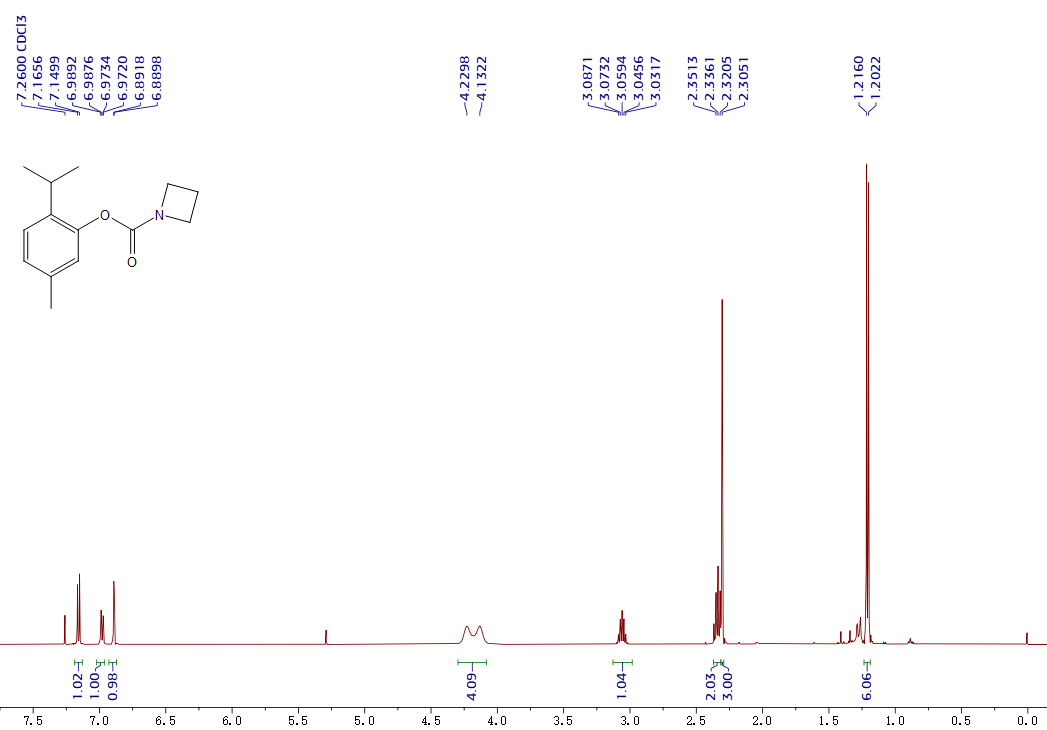
***

***
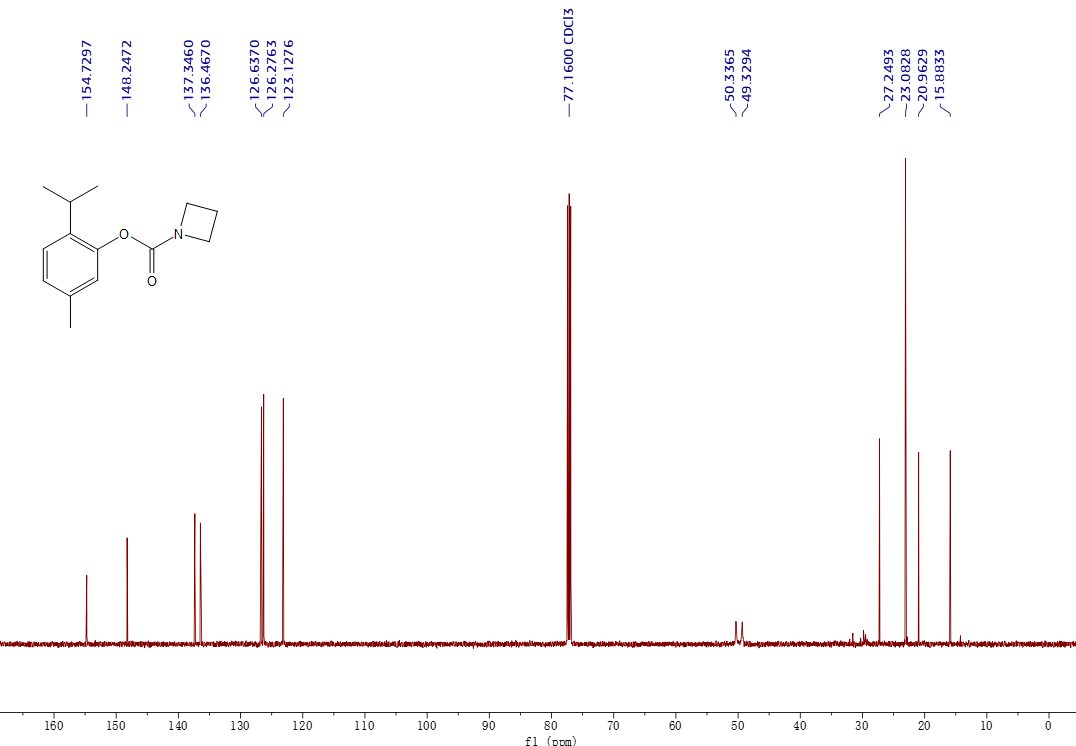
*
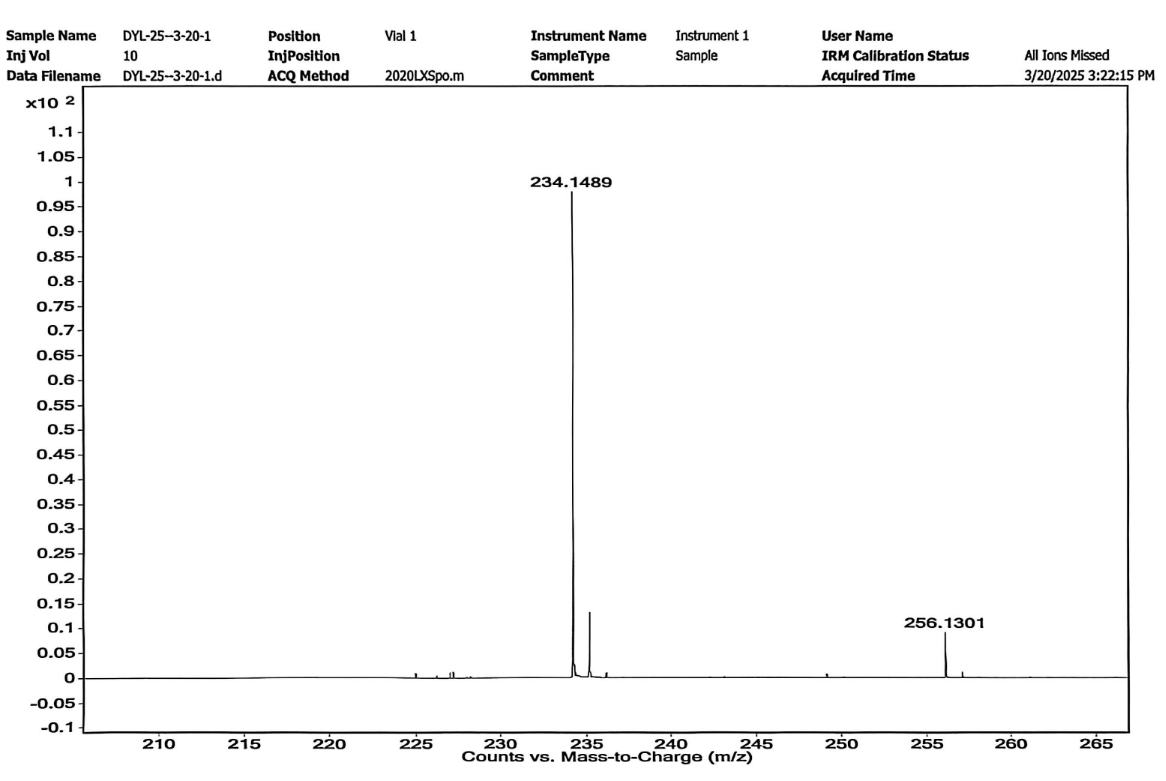

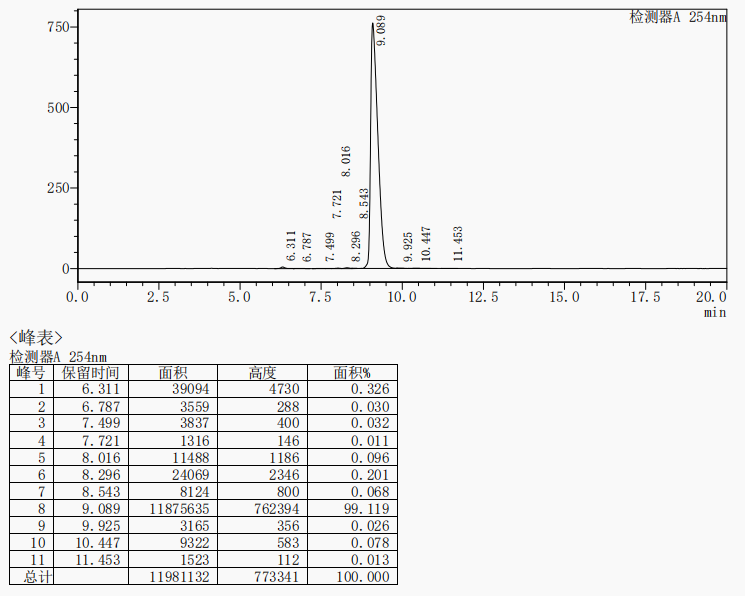
Figure S4.** ^1^H-NMR, ^13^C-NMR, HRMS (ESI) and HPLC spectra of compound **TC-4.**

***
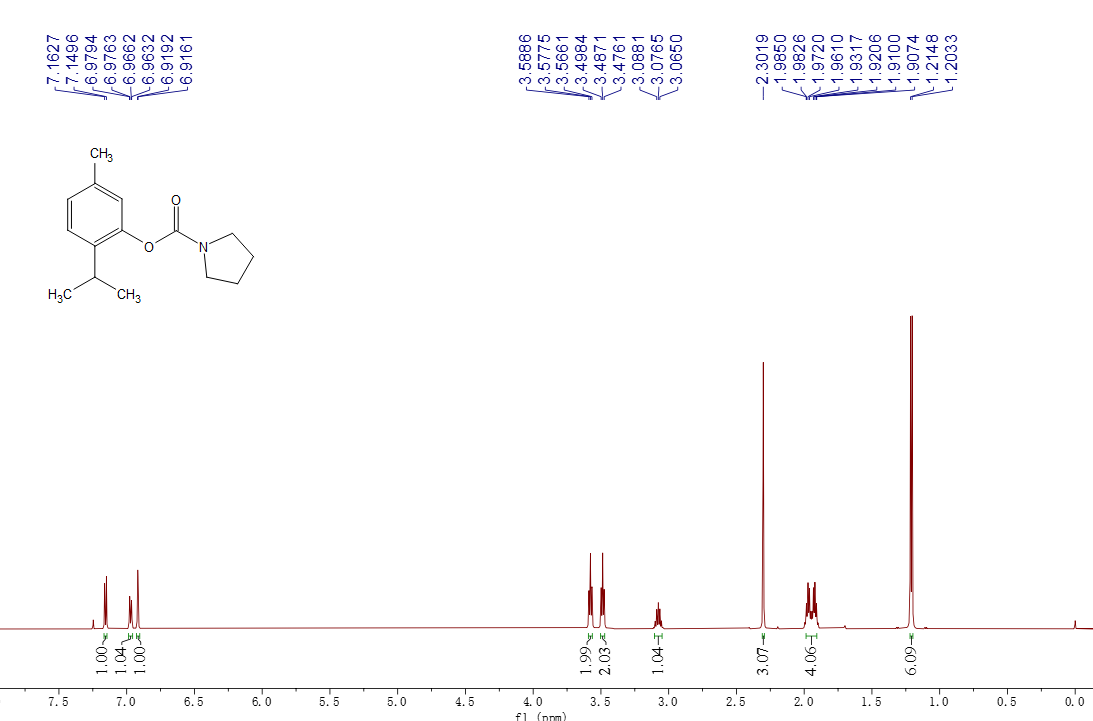
***

***
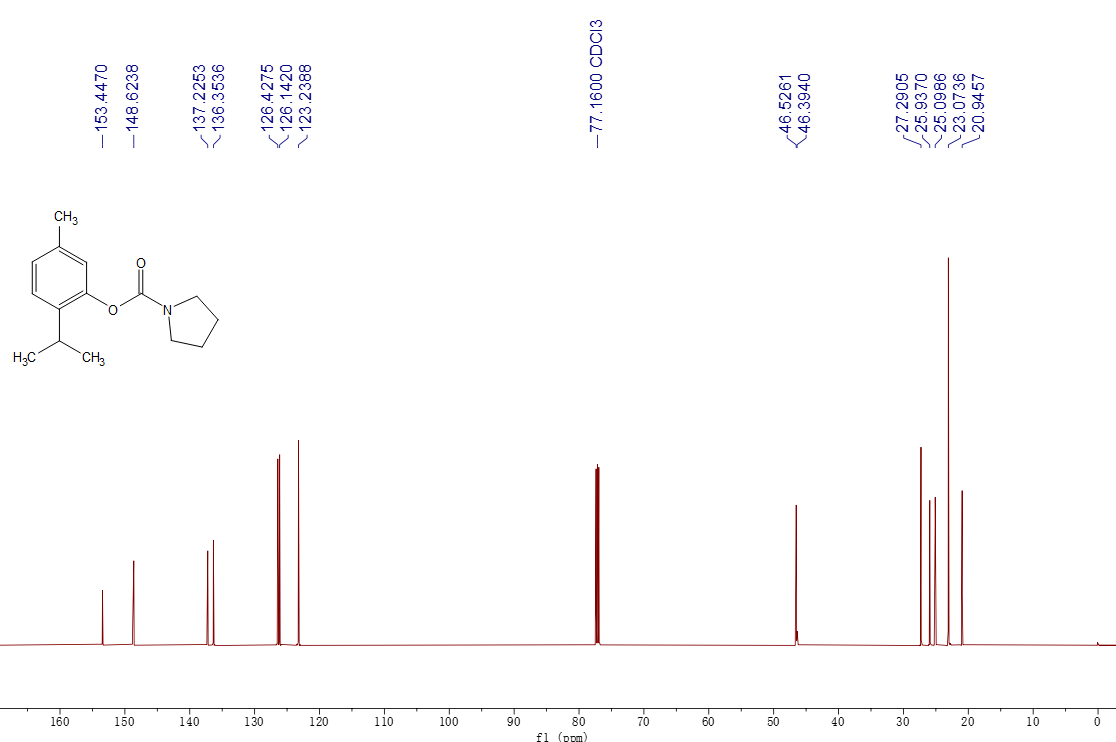
*
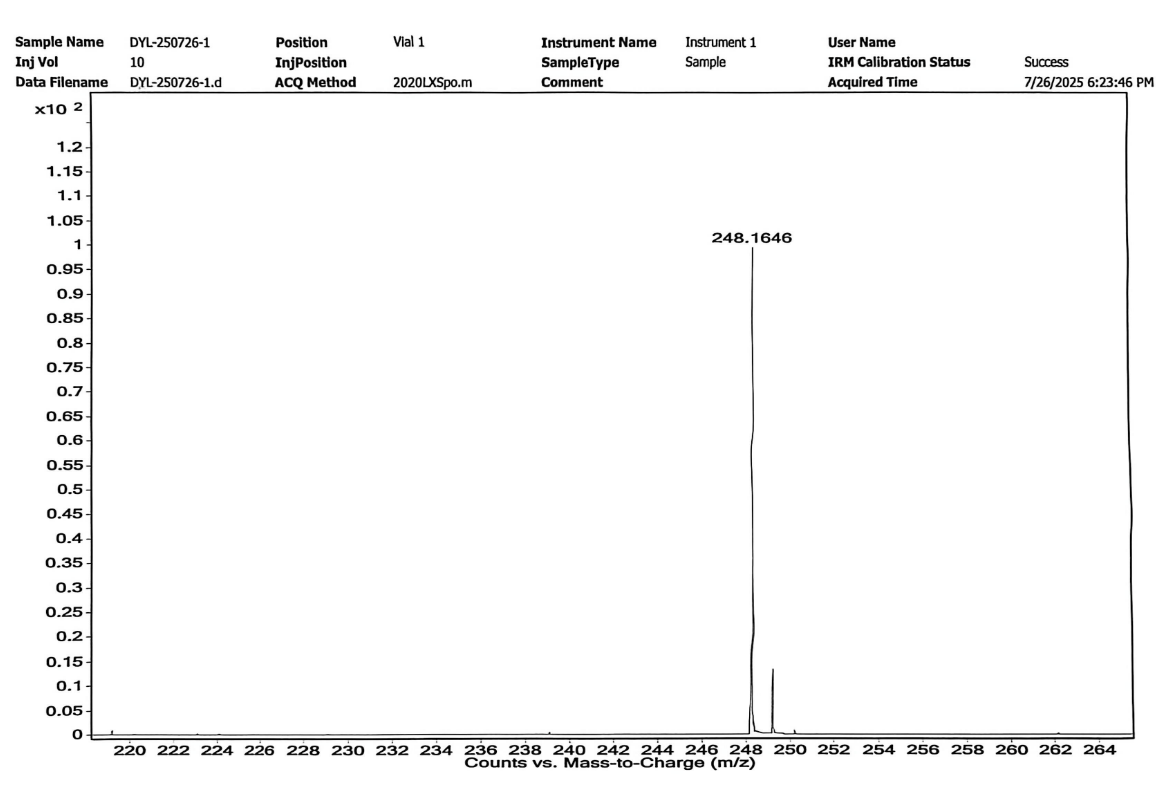

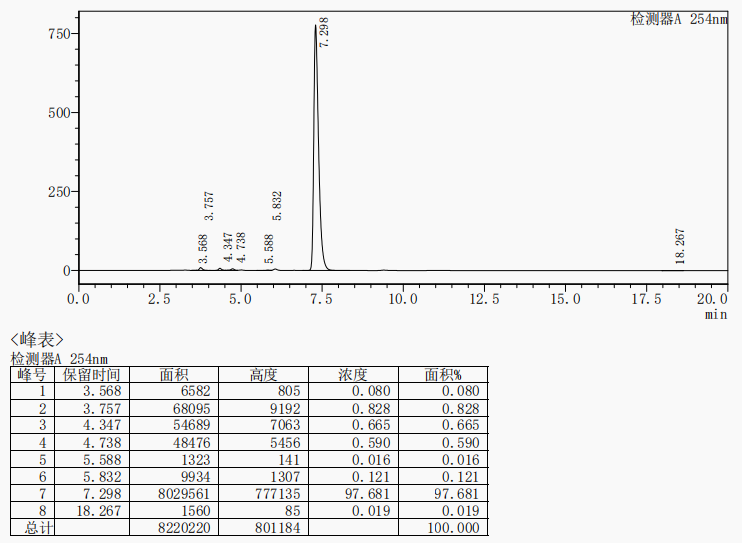
Figure S5.** ^1^H-NMR, ^13^C-NMR, HRMS (ESI) and HPLC spectra of compound **TC-5.**

***
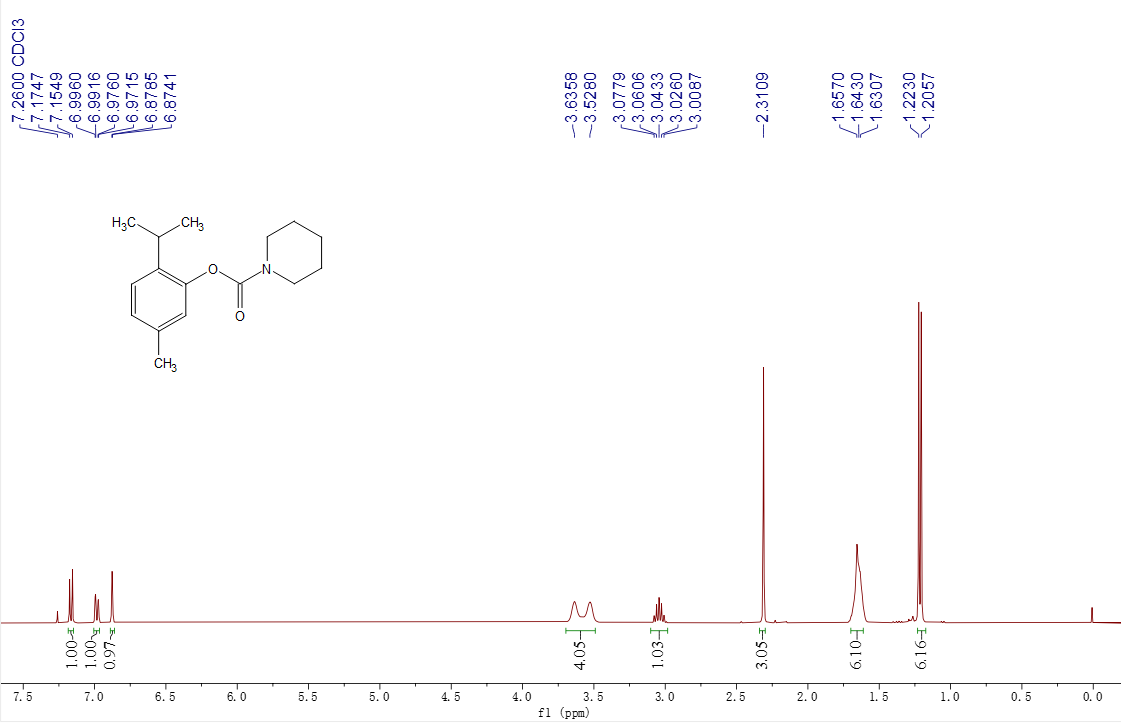

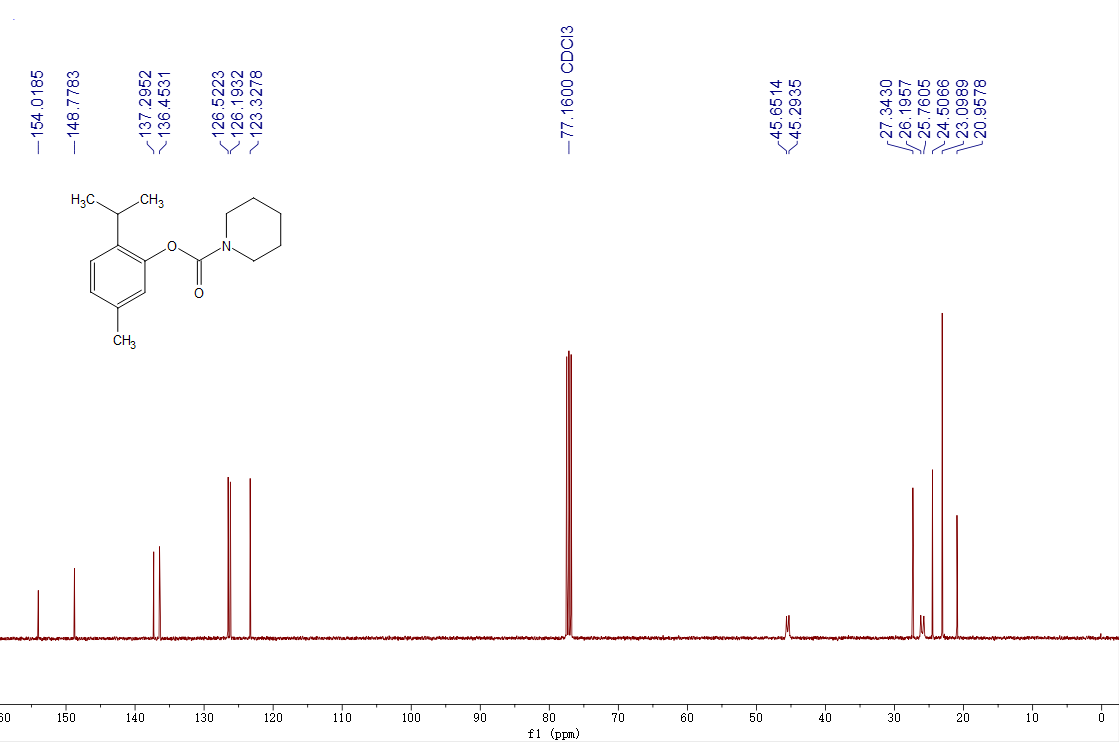
*** ***
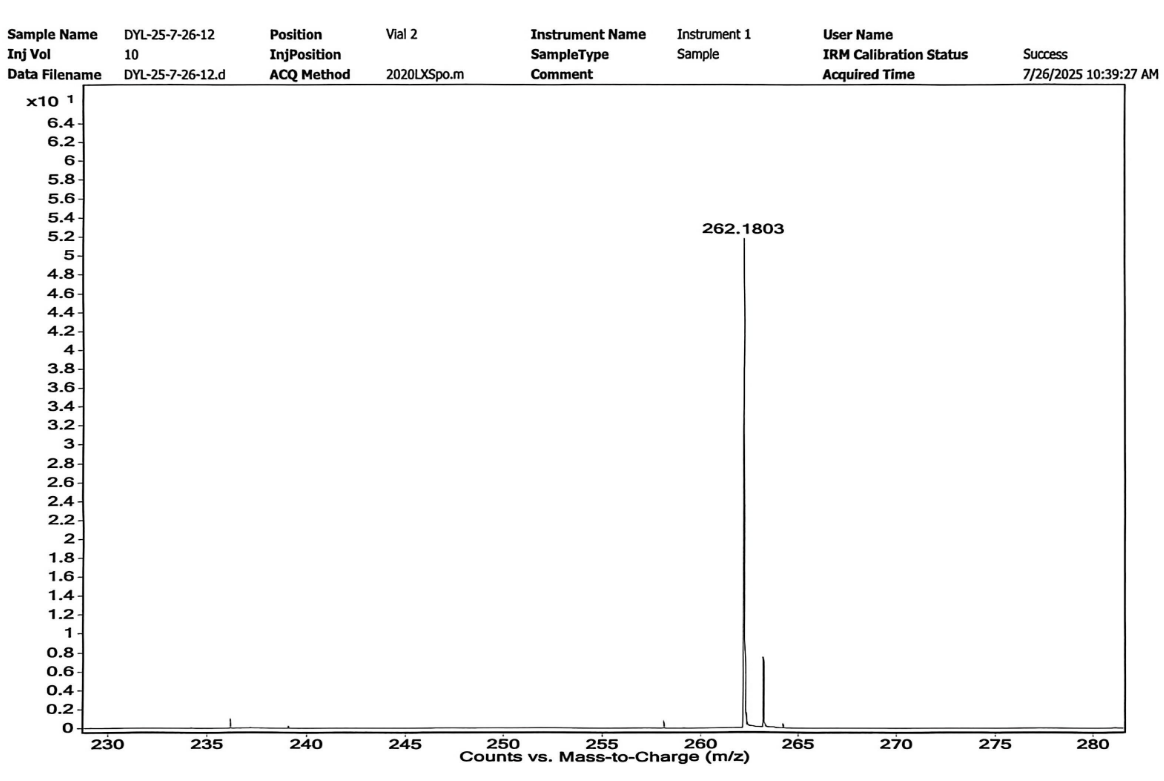
***
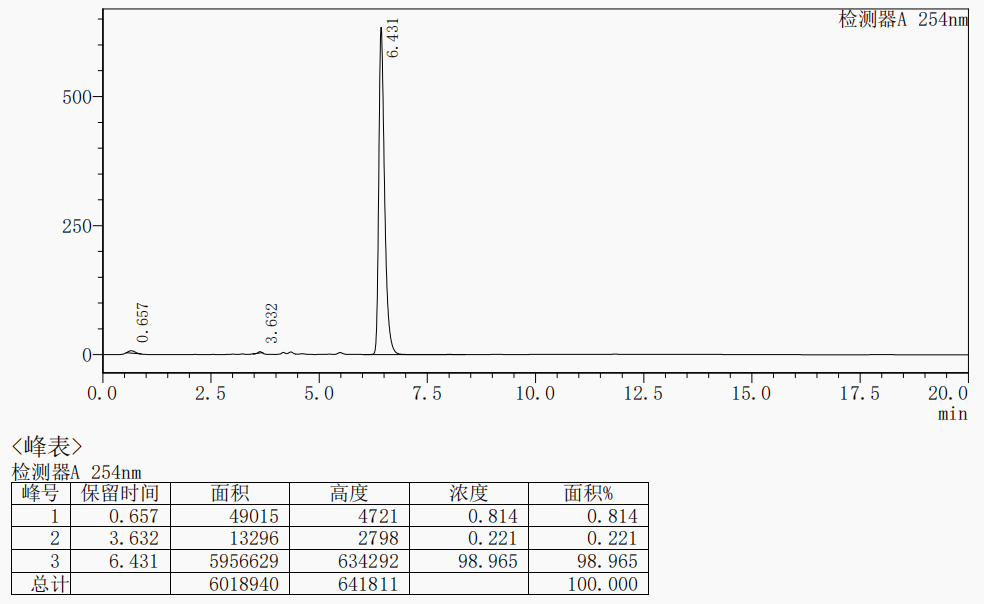


**Figure S6.** ^1^H-NMR, ^13^C-NMR, HRMS (ESI) and HPLC spectra of compound **TC-6.**

**
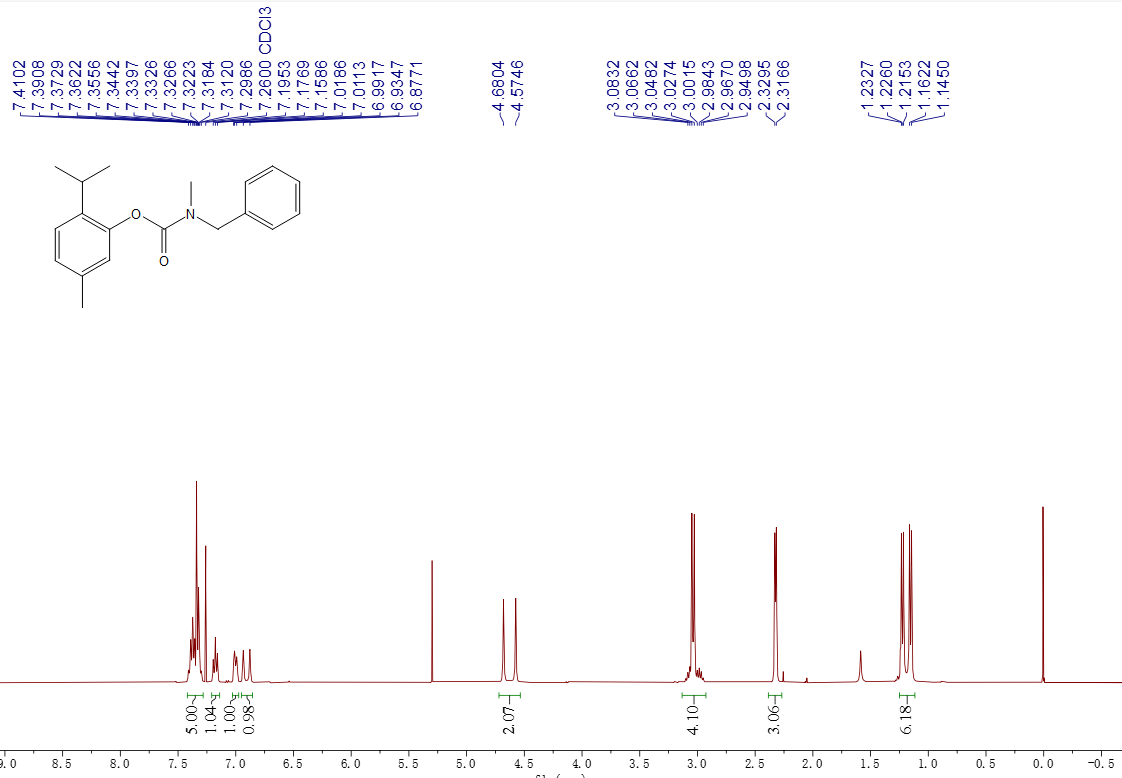
** **
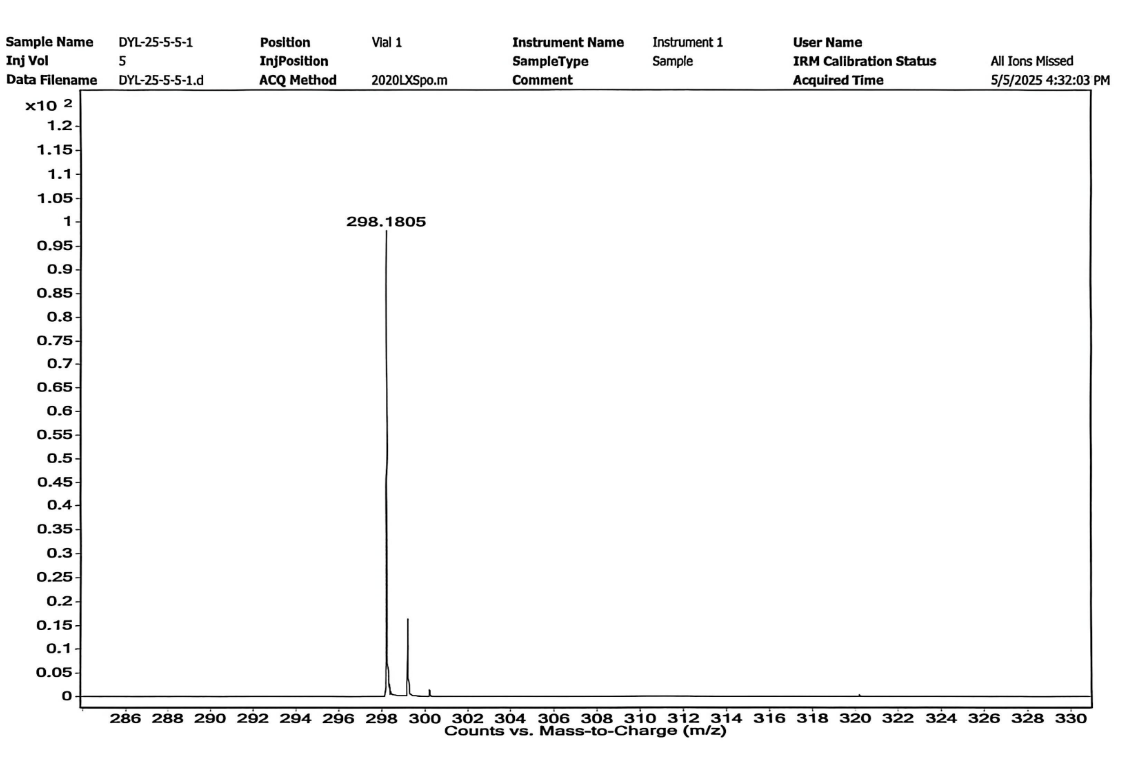
**
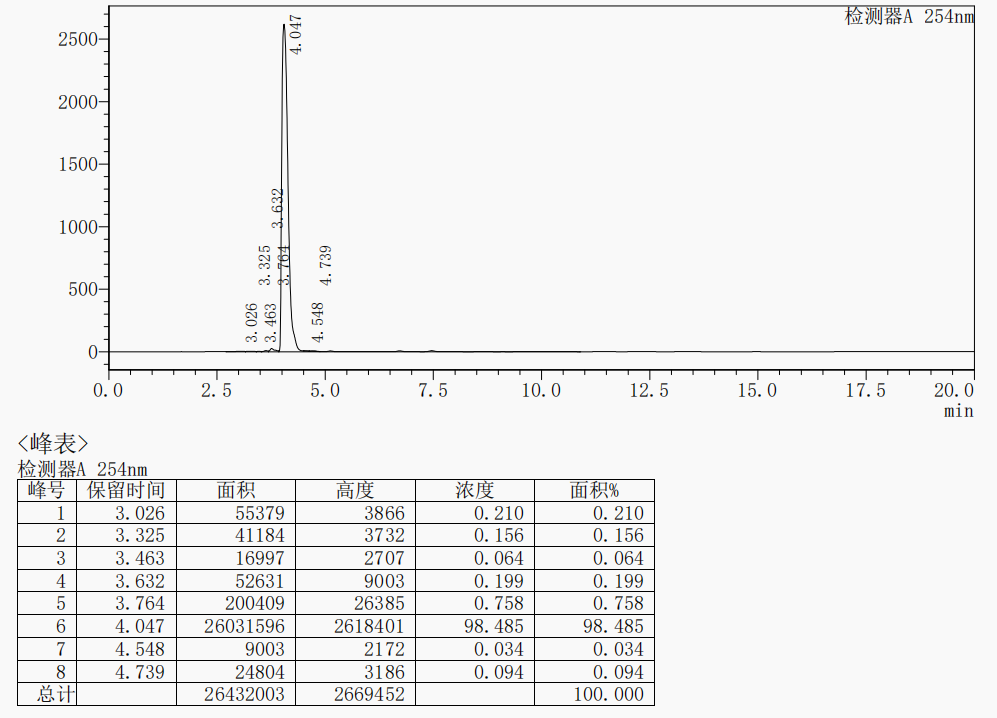


**Figure S7.** ^1^H-NMR, HRMS (ESI) and HPLC spectra of compound **TC-7.**

**
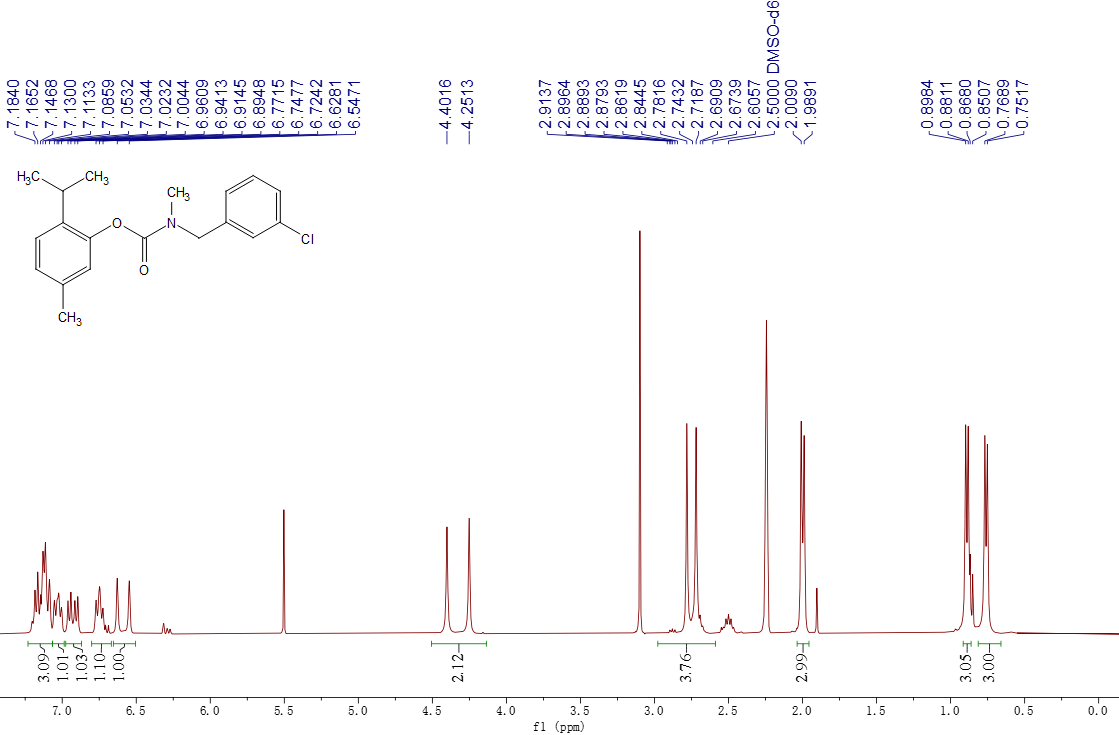

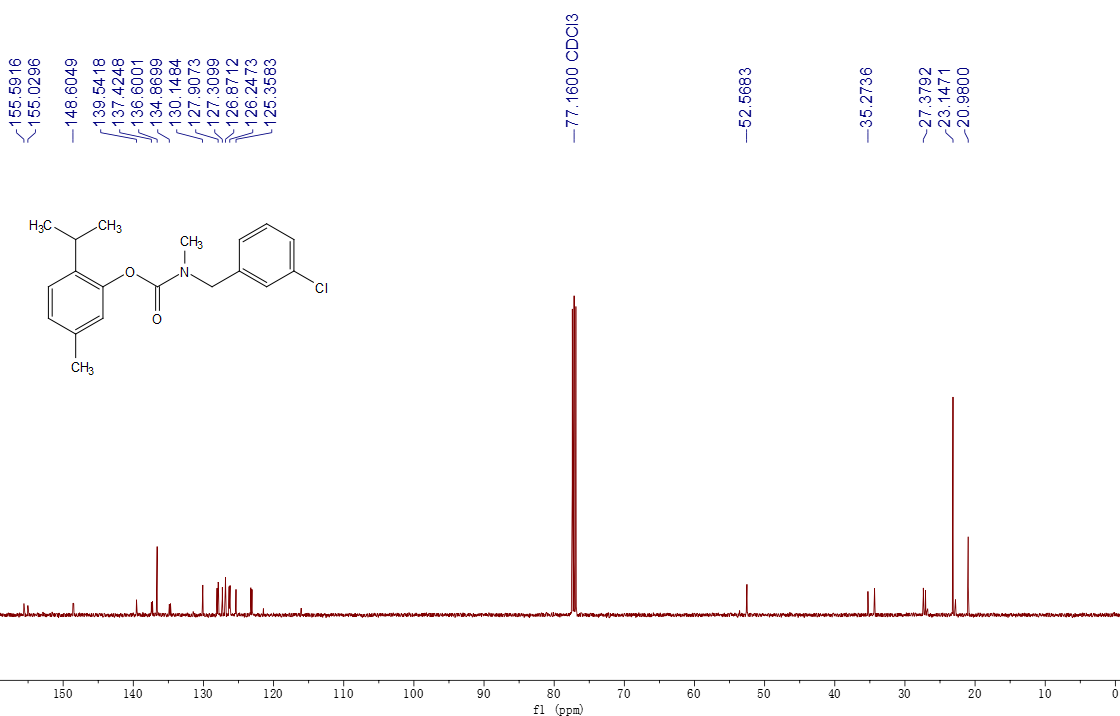
** **
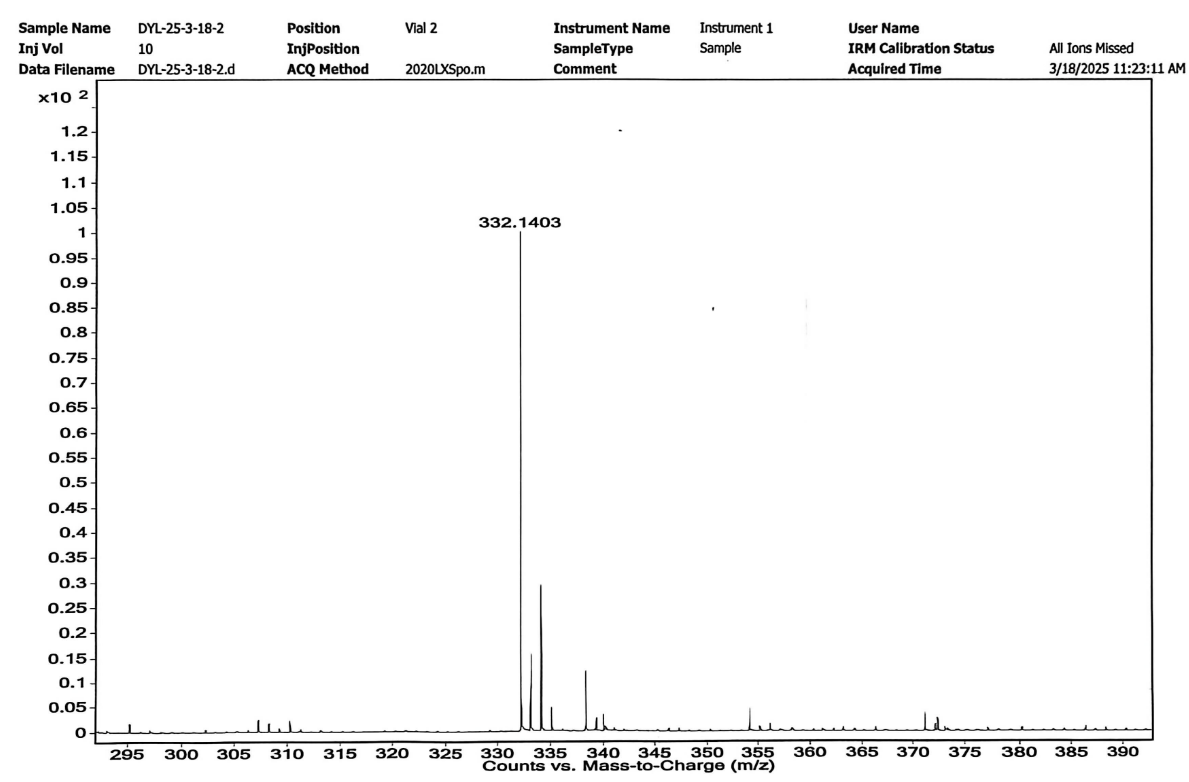
**
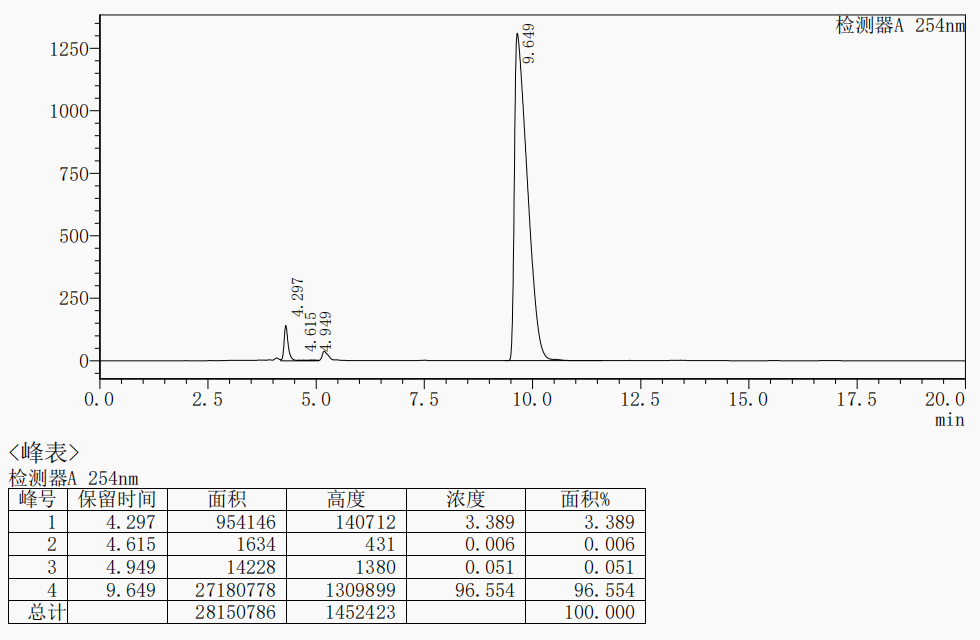


**Figure S8.** ^1^H-NMR, ^13^C-NMR, HRMS (ESI) and HPLC spectra of compound **TC-8.
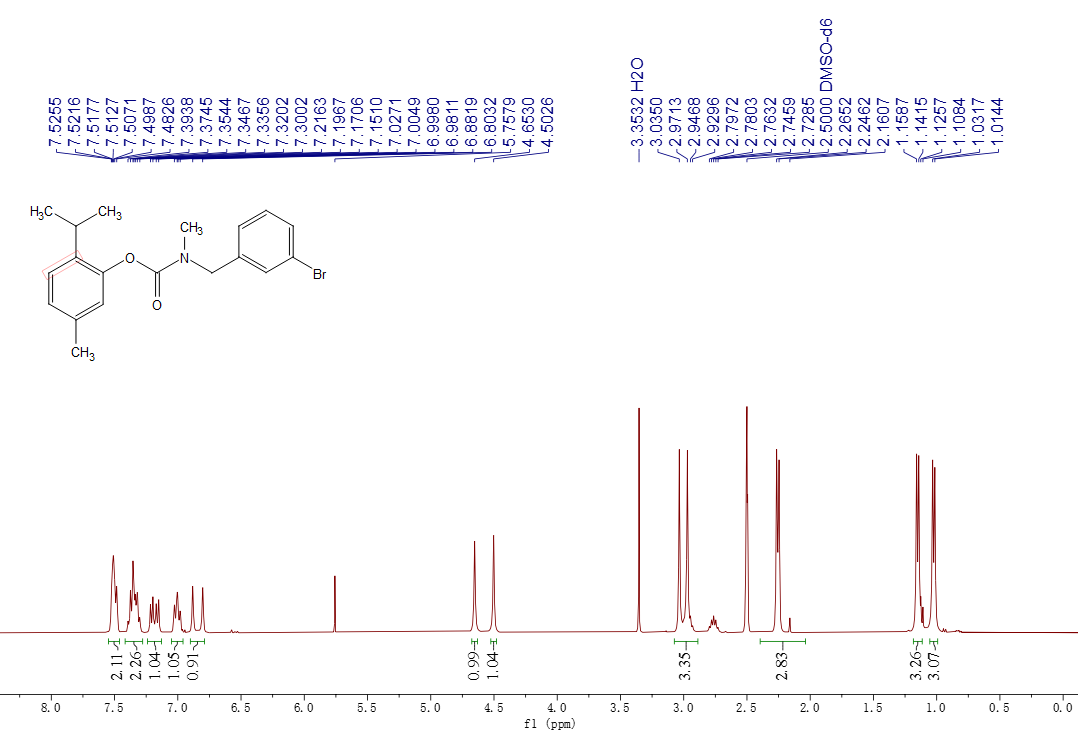
**

**
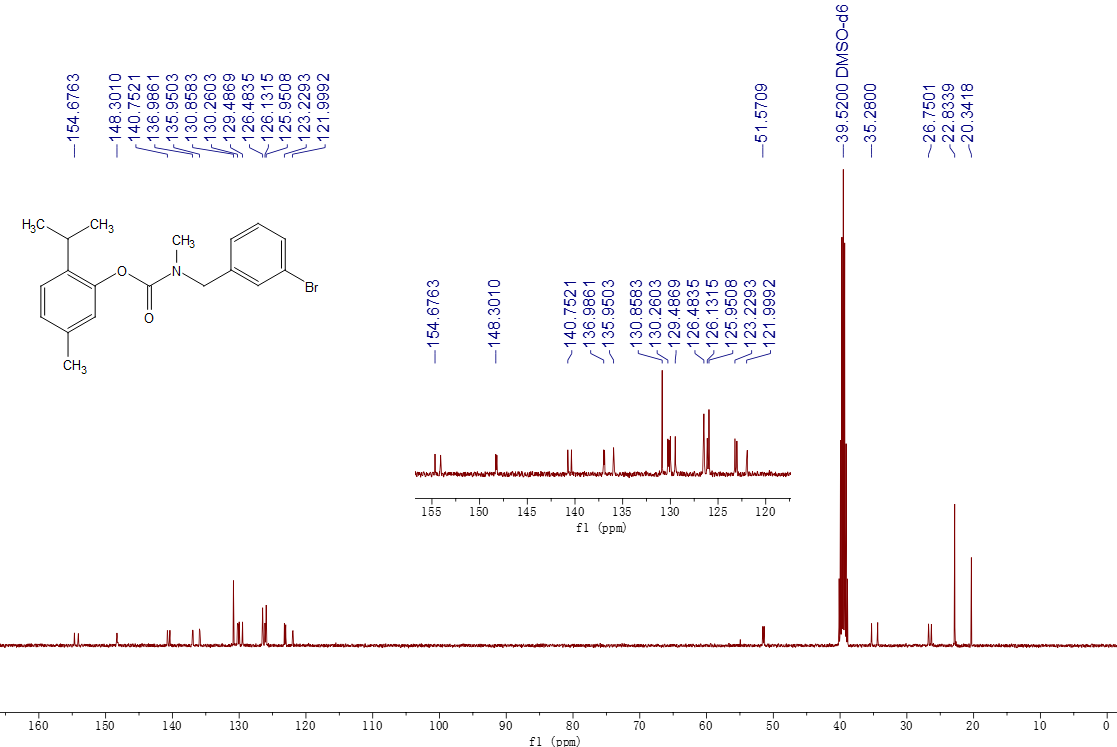

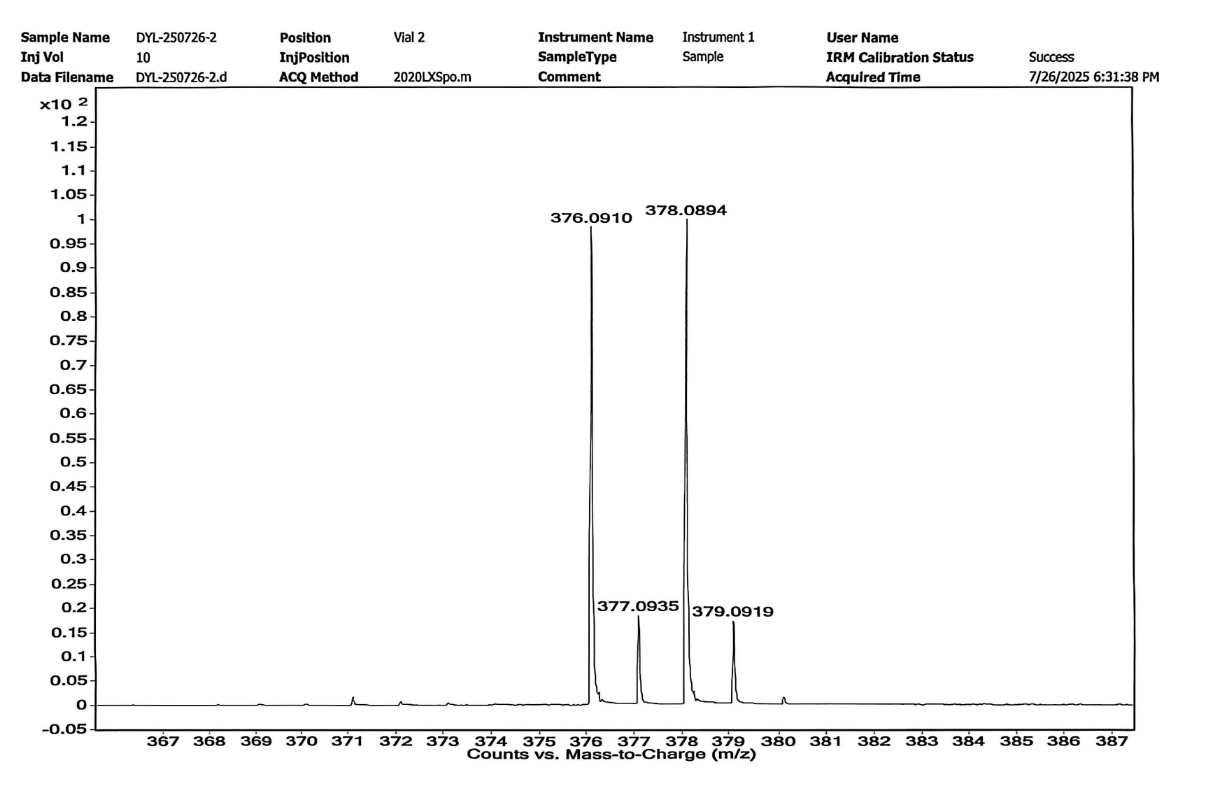

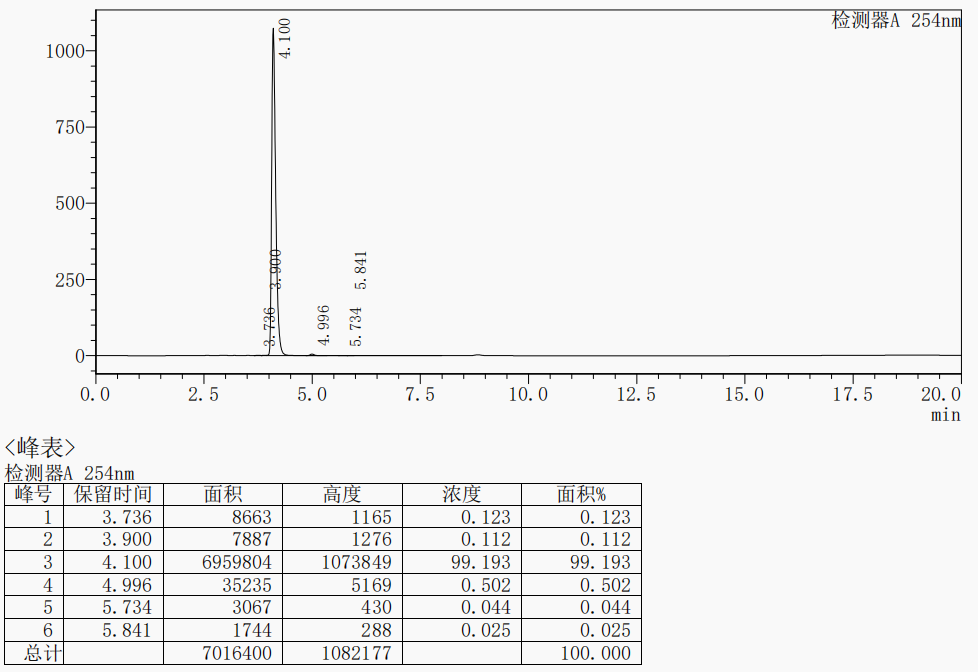
Figure S9.** ^1^H-NMR, ^13^C-NMR, HRMS (ESI) and HPLC spectra of compound **TC-9.**

***
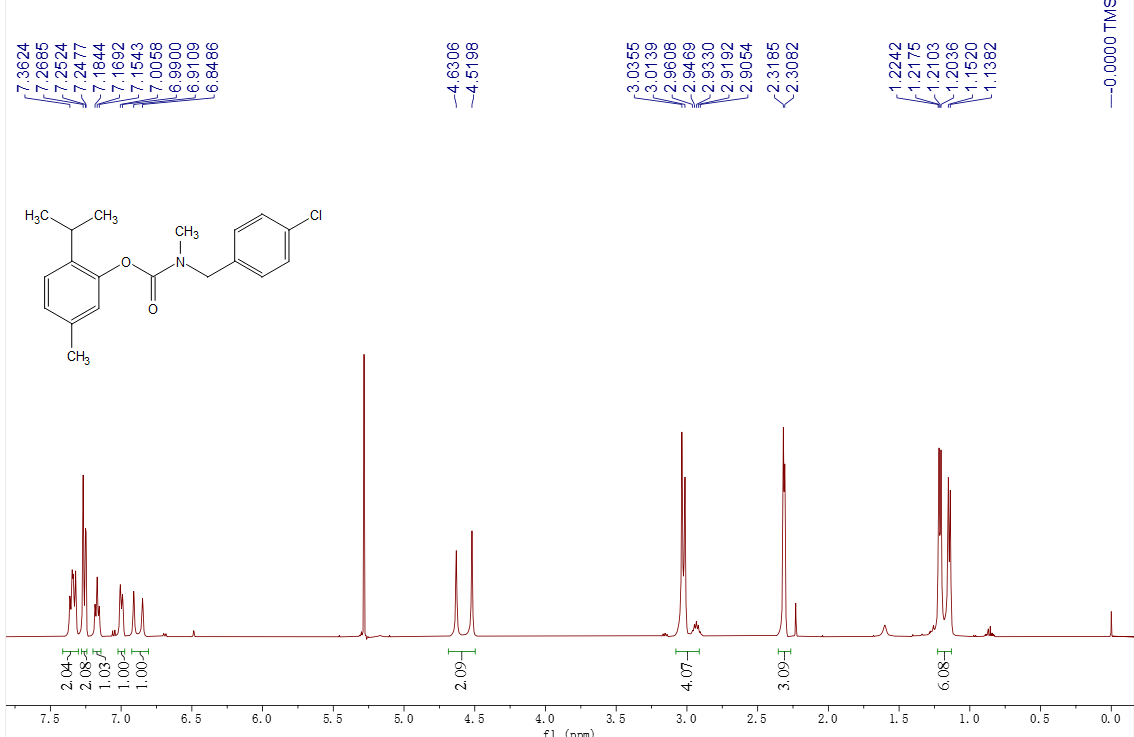
***

***
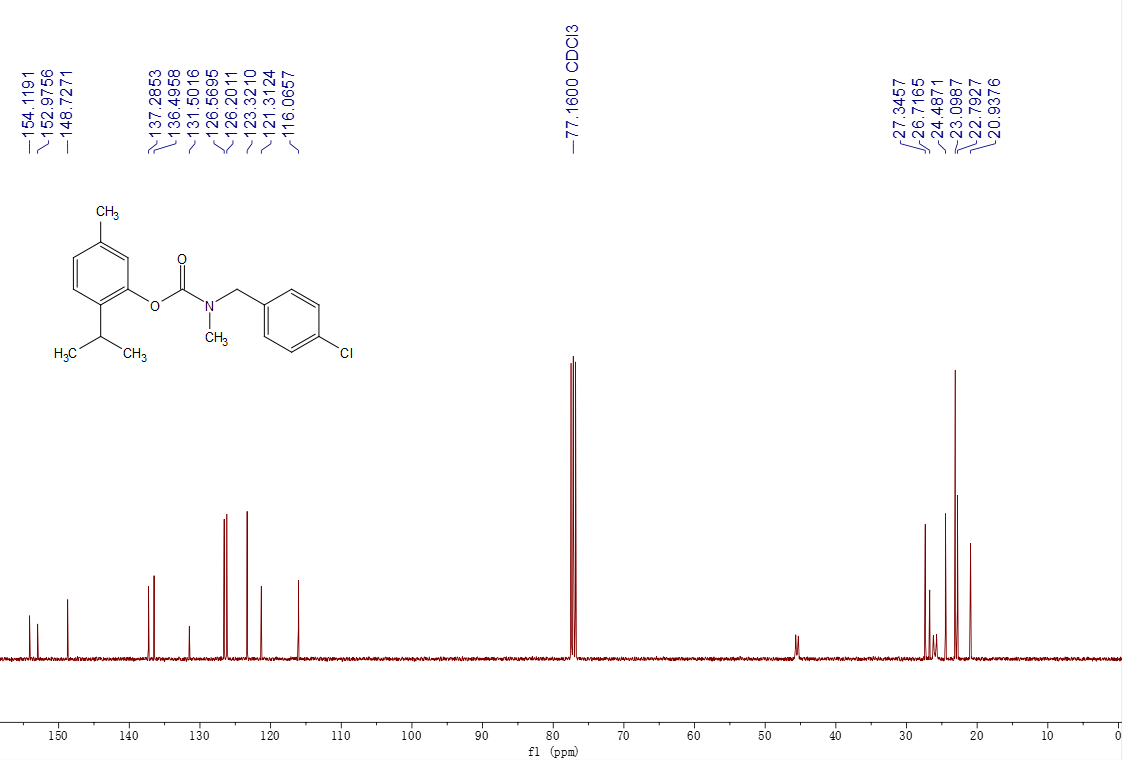
*
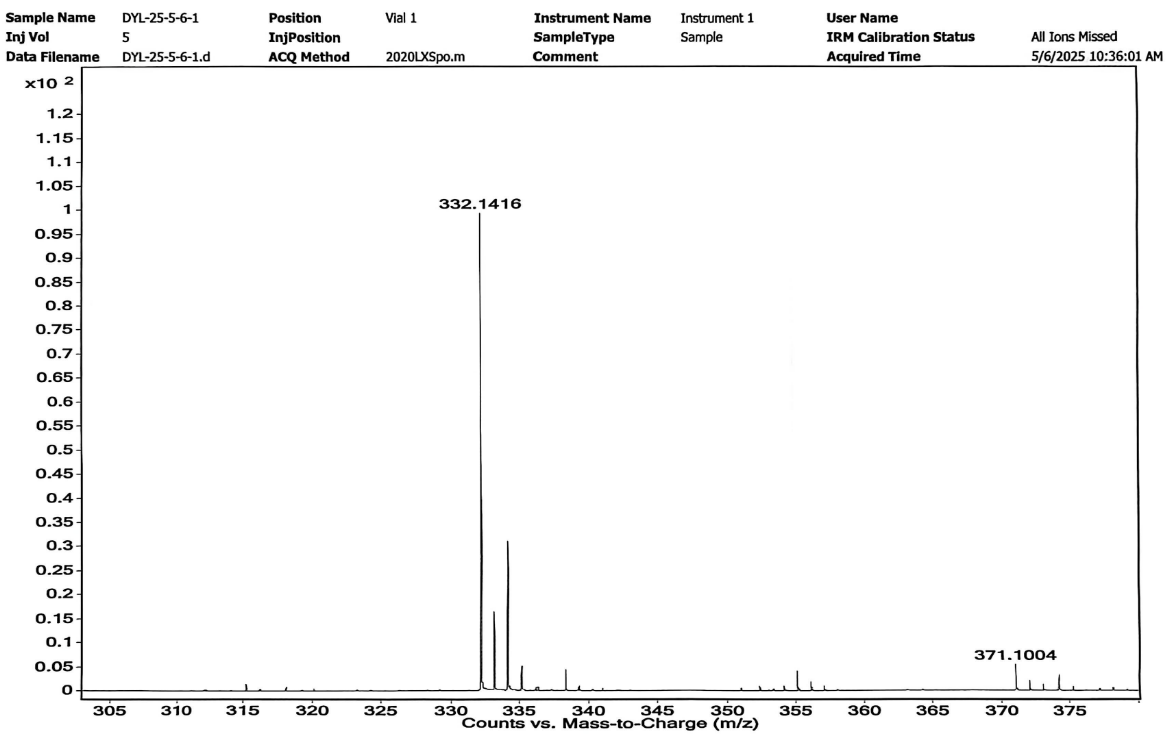

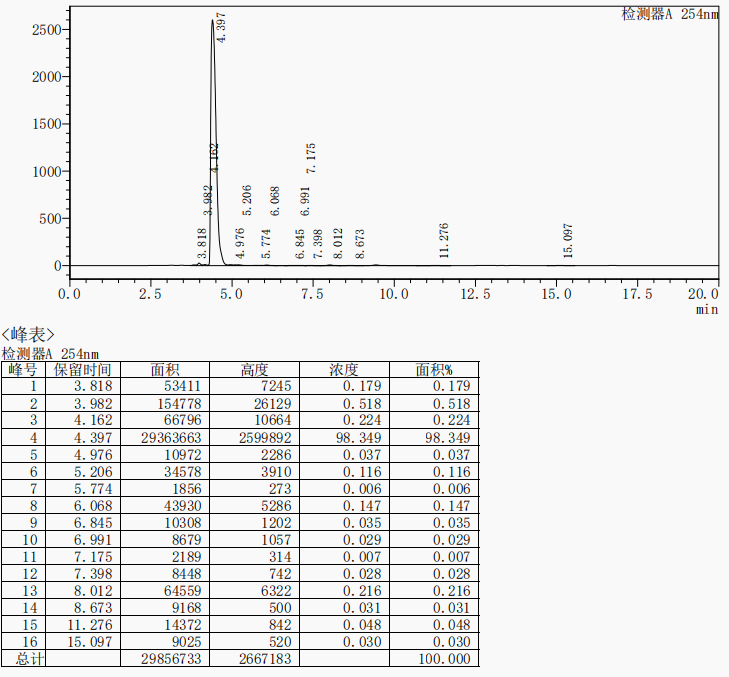
Figure S10.** ^1^H-NMR, ^13^C-NMR, HRMS (ESI) and HPLC spectra of compound **TC-10.**

***
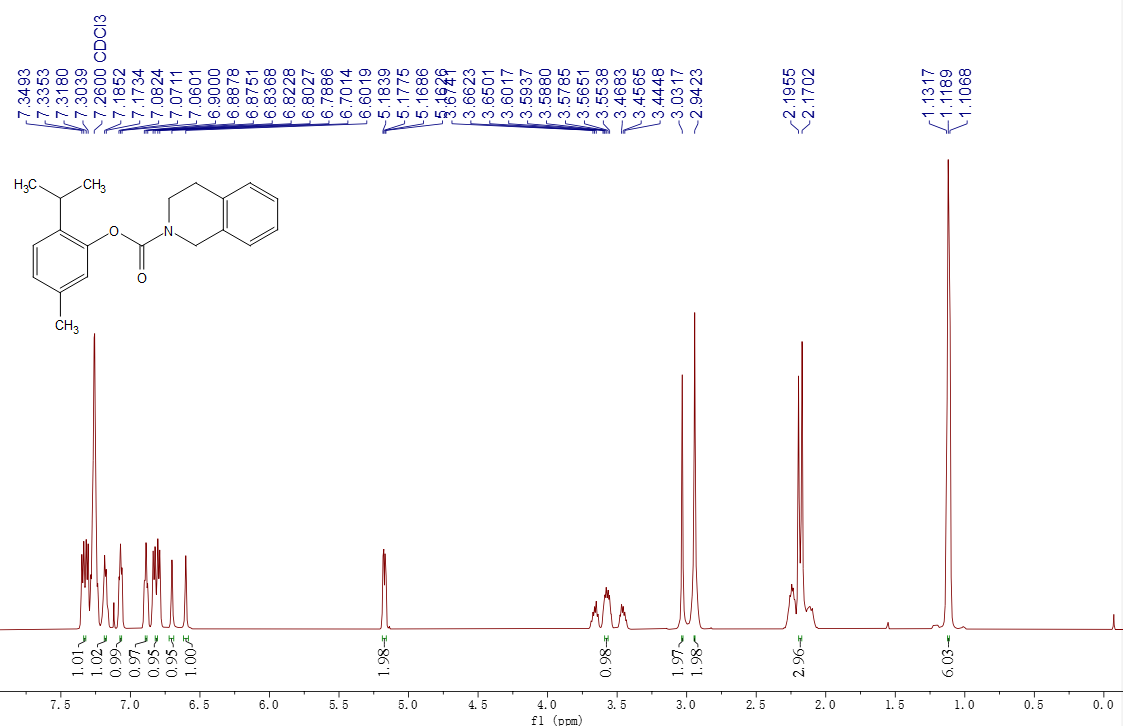
***

***
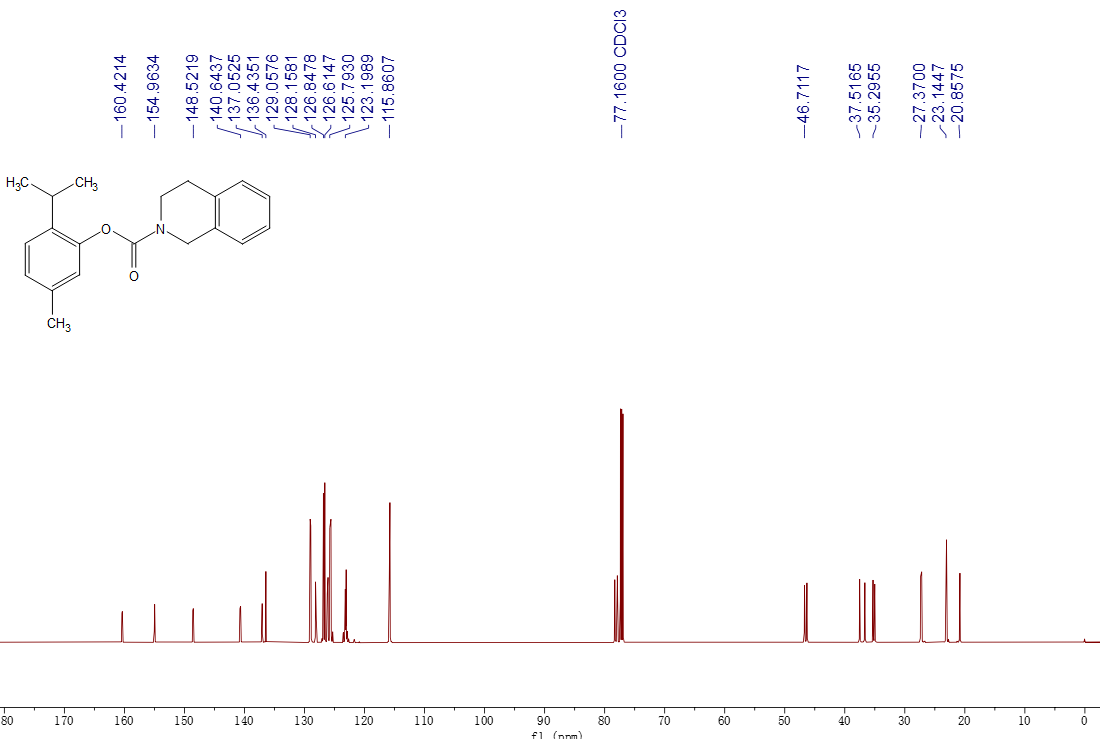
*
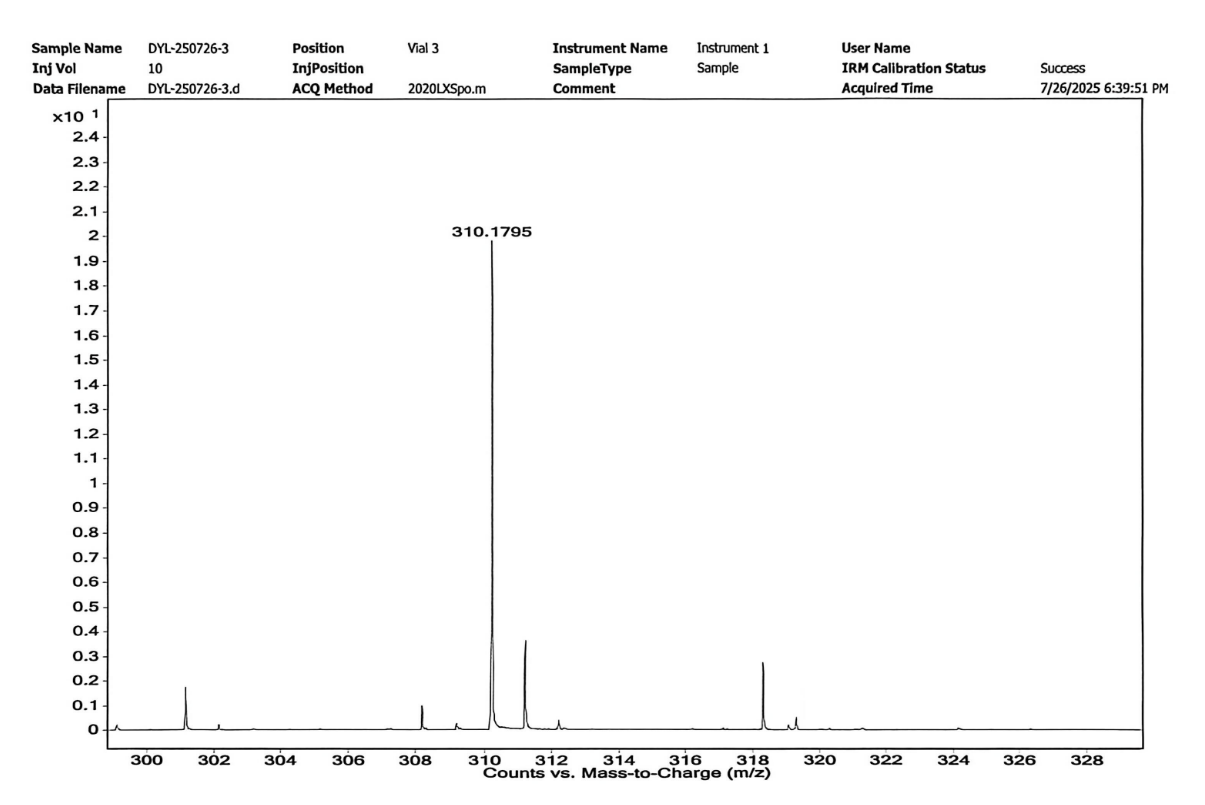

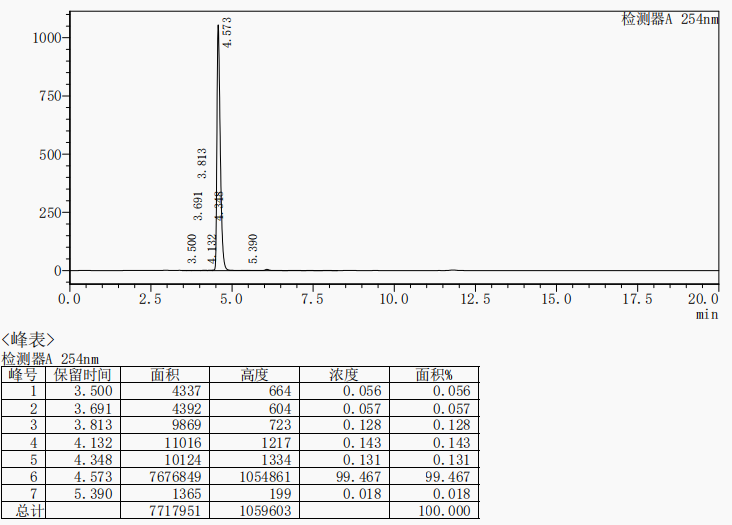
Figure S11.** ^1^H-NMR, ^13^C-NMR, HRMS (ESI) and HPLC spectra of compound **TC-11.**

***
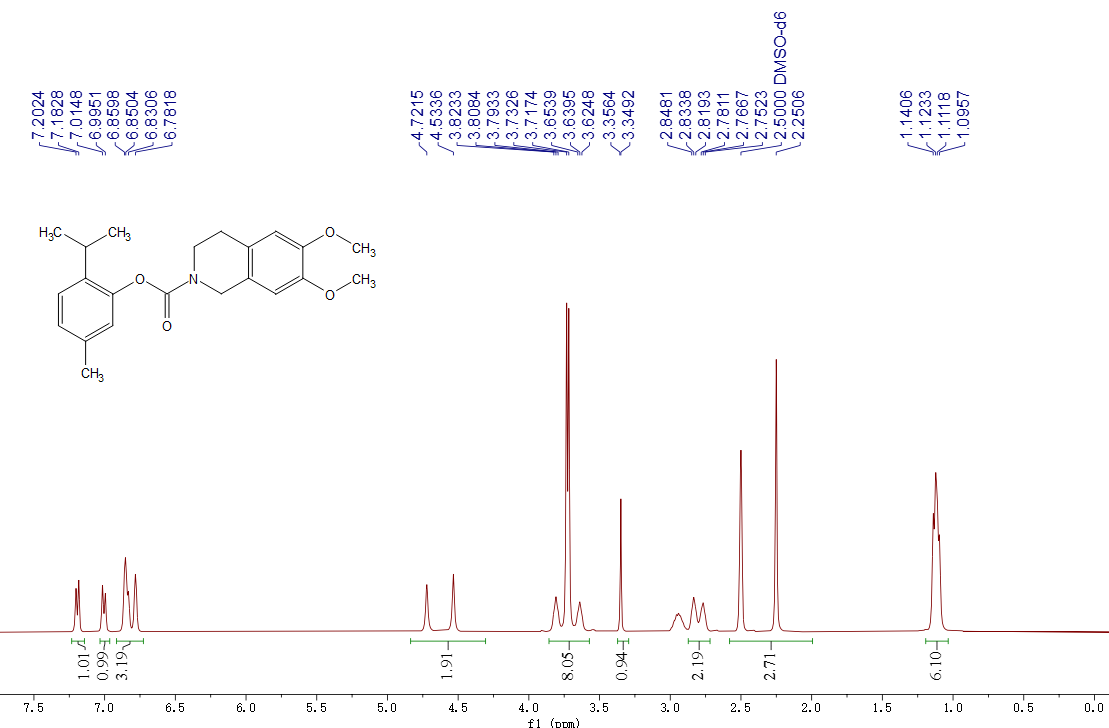

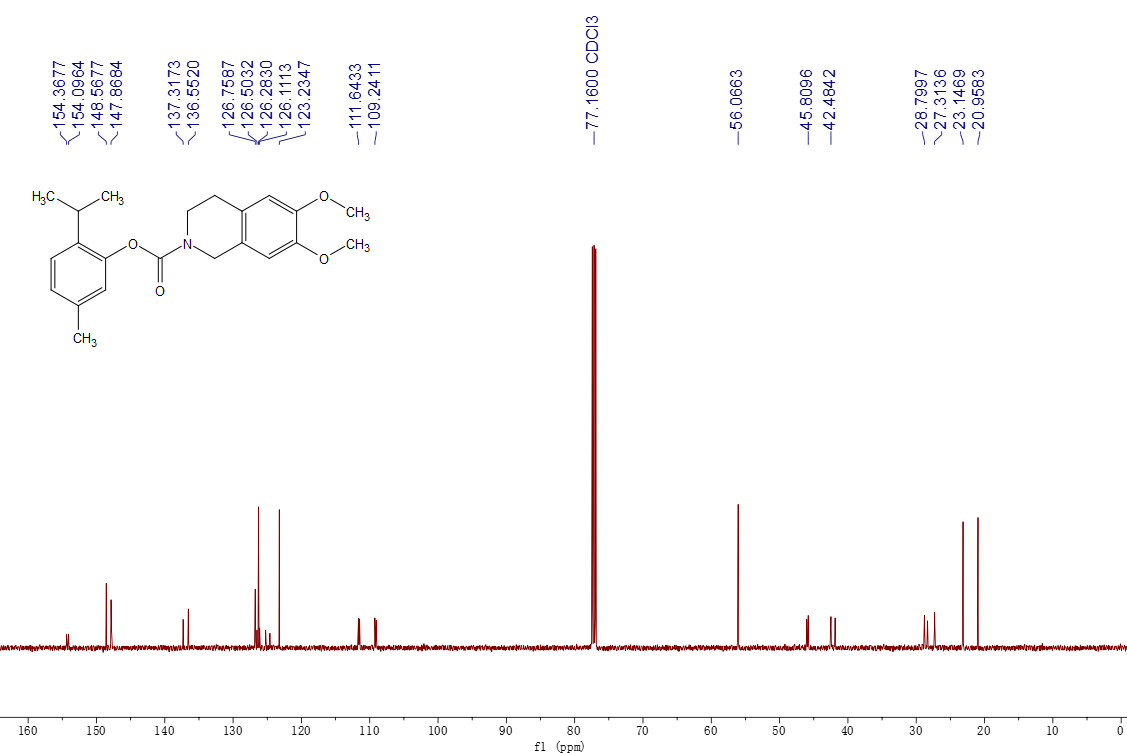
*** ***
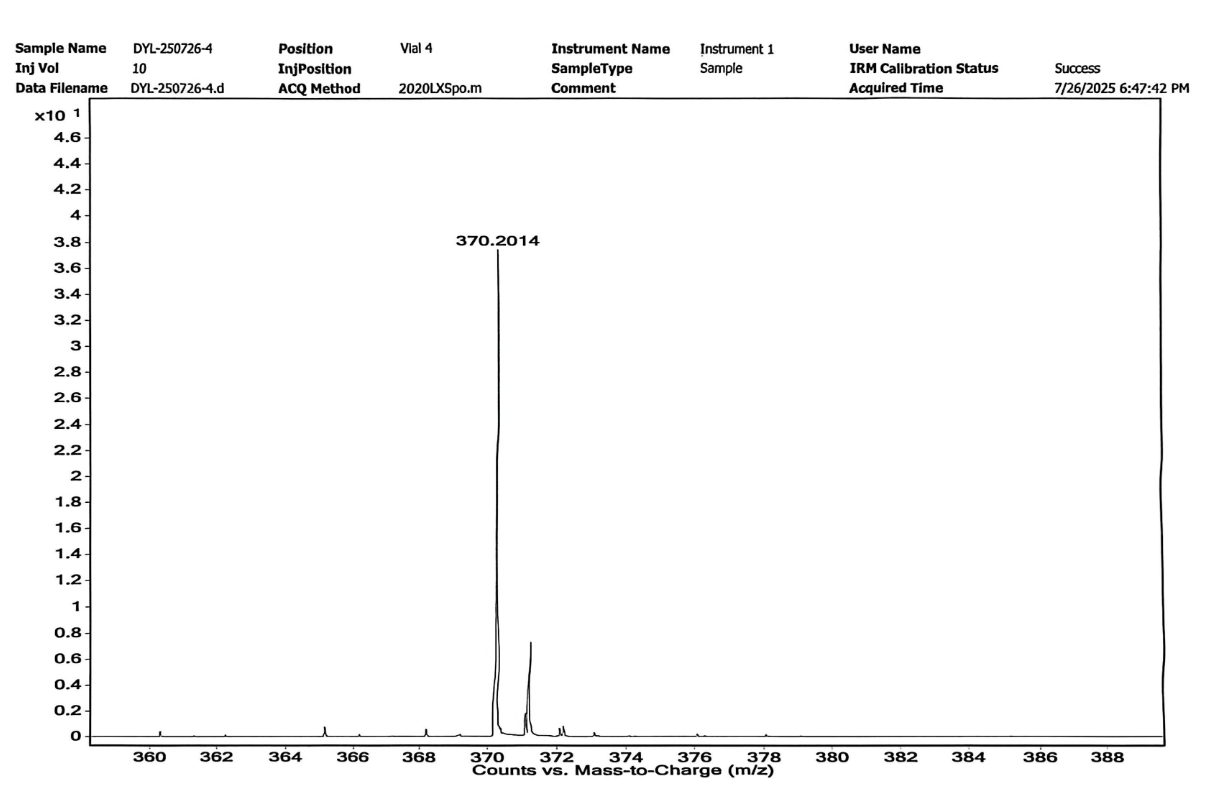
***
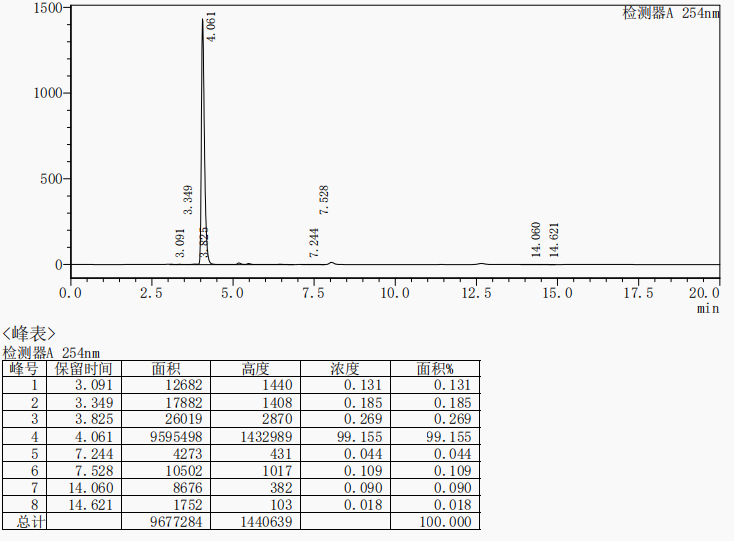


**Figure S12.** ^1^H-NMR, ^13^C-NMR, HRMS (ESI) and HPLC spectra of compound **TC-12.**
